# Arbuscular Mycorrhizal Fungi Alleviate Cadmium Phytotoxicity by Regulating Cadmium Mobility, Physiological Responses, and Gene Expression Patterns in *Malus hupehensis* Rehd

**DOI:** 10.3390/ijms26041418

**Published:** 2025-02-07

**Authors:** Xiaolei Zhuang, Siyu Liu, Shengzhe Xu, Sijun Qin, Deguo Lyu, Jiali He, Jiangtao Zhou

**Affiliations:** 1 College of Horticulture, Shenyang Agricultural University, Shenyang 110866, China; 2 Key Lab of Fruit Quality Development and Regulation of Liaoning Province, Shenyang 110866, China; 3 Research Institute of Pomology, Chinese Academy of Agricultural Sciences, Xingcheng 125100, China

**Keywords:** cadmium, arbuscular mycorrhizal fungi, orchard, reactive oxygen species, soil pollution

## Abstract

Arbuscular mycorrhizal fungi (AMF) affect cadmium (Cd) accumulation and tolerance in host plants. However, the effects of AMF on Cd accumulation and phytotoxicity and their underlying mechanism in apples remain uncharacterized. In this study, the comprehensive physiological and molecular responses of uninoculated and *Rhizophagus intraradices*-inoculated *Malus hupehensis* Rehd. rootstocks exposed to 0 or 300 μM Cd were investigated. AMF inoculation mitigated Cd-induced growth and photosynthesis inhibition and nutrient ion disorders. It also lowered the concentrations of Cd in all tissues and reduced Cd transport to the shoots. Compared to uninoculated apple plants, those inoculated with mycorrhizal fungi reduced the mobility and toxicity of Cd by altering its form and binding it to the cell walls of the roots and leaves. AMF inoculation ameliorated Cd stress by altering endogenous phytohormone levels and triggering enzymatic and non-enzymatic antioxidant systems. Transcriptome analysis revealed that the differentially expressed genes (DEGs) associated with AMF under Cd stress regulated carbohydrate and amino acid biosynthesis and metabolism, as well as phytohormone biosynthesis and signal transduction. Furthermore, AMF inoculation downregulated certain genes involved in Cd uptake and transport while upregulating other genes involved in detoxification. These results suggest that AMF alleviate Cd phytotoxicity by orchestrated physiological and transcriptomic regulation in *M. hupehensis* Rehd., providing valuable insights into the efficacy of AMF inoculation in improving the heavy metal resistance of fruit trees.

## 1. Introduction

Cadmium (Cd) is a toxic heavy metal which damages plants even at low concentrations [1]. Li et al. [2] found that the Cd concentrations in certain apple orchard soils of the Northeast Jiaodong Peninsula in China exceeded the 0.3 mg kg^−1^ secondary limit set in the Chinese soil environmental quality standard. In the Liaodong Peninsula, Northeast China, the Cd content in 6.34% of all apple peels sampled surpassed the national maximum permissible level of 0.03 mg kg^−1^ dry weight (DW) [3]. Cd has a high solubility, strong mobility, and a long biological half-time, and it is readily absorbed by plant roots [4,5]. High Cd concentrations disrupt various biochemical and physiological processes and inhibit growth in plants [6,7,8]. The Cd that plants absorb may be enriched and transferred to the food chain, where it can threaten human health [9]. Therefore, Cd accumulation and toxicity in plant tissues must be mitigated.

Arbuscular mycorrhizal fungi (AMF) are ubiquitous rhizosphere microorganisms that establish beneficial symbiotic (mutualistic) relationships with ~80% of all terrestrial plants [10]. AMF facilitate nitrogen and phosphorus uptake in their host plants, which in turn supply the fungi with sugars and lipids [11,12,13]. AMF also regulate Cd accumulation, translocation, and tolerance in host plants [8,14]. Depending on the host species and fungal isolates, AMF may increase or decrease plant Cd accumulation and translocation. For example, *Rhizophagus irregularis* (*R. irregularis*) inoculation significantly increased Cd accumulation in the roots but reduced Cd transport to the stems of *Phragmites australis* (Cav.) Trin. ex Steud. [15]. In contrast, Liu et al. [14] reported that *Funneliformis mosseae* inoculation inhibited Cd accumulation in the roots, stems, and leaves but promoted root-to-shoot Cd translocation in *Populus yunnanensis*. As host/fungus-dependent processes may account for these discrepancies, the present study aimed to determine how AMF affect Cd accumulation and tolerance in apple trees.

AMF affect plant Cd accumulation and tolerance by several mechanisms. First, AMF enhance nutrient and water uptake in host plants by expanding their root absorption area and, by extension, promoting their growth [8]. Second, AMF increase the concentrations of osmoregulatory substances, improve antioxidant capacity, stabilize cell membranes, and alleviate Cd-induced oxidative stress in host plants [8,14,16]. Third, AMF lower the toxicity of Cd by regulating its subcellular distribution and chemical forms and altering the cell wall components in host plants [15,17,18]. Fourth, AMF regulate transcriptomic responses and, by extension, the absorption, accumulation, and tolerance of Cd in host plants. For example, in the roots of mycorrhizal *P. yunnanensis* under Cd stress, the DEGs were associated mainly with antioxidant activity and metal cation transport, compartmentalization, and chelation [14]. You et al. [19] showed that *F. mosseae* enhanced Cd absorption in *Phragmites australis* by upregulating zinc transporter (ZIP) and natural resistance-associated macrophage protein (NRAMP) family genes. *R. irregularis* increased metal flux out of cells and/or into vacuoles by upregulating the ABC family genes in its host *Broussonetia papyrifera* under Cd stress [20]. The comprehensive expression profiles of the genes involved in Cd uptake, translocation, and detoxification must be characterized to elucidate the molecular mechanisms by which AMF influence Cd accumulation and tolerance in host plants. The physiological and molecular mechanisms by which AMF affect Cd accumulation and tolerance have been investigated in *P. yunnanensis* [14], *Medicago sativa* L. [8], and *B. papyrifera* [20]. To the best of our knowledge, however, the comprehensive physiological and molecular mechanisms by which AMF regulate Cd accumulation and tolerance in woody fruit trees have not been examined.

Apple is a widely cultivated fruit crop in China and worldwide, and it has important economic and nutritional value [21]. As Cd pollution is increasing in apple orchards [2], effective measures are urgently needed to improve the tolerance and reduce the absorption and transport of Cd in apple trees. *Malus hupehensis* Rehd. is a unique germplasm resource in China. This apomictic variety is commonly used as a rootstock for apple propagation but has low Cd tolerance [7]. *Rhizophagus intraradices* (*R. intraradices*) enhance the tolerance and modulate the absorption and transport of Cd in various host plants [22,23]. In addition, it has been found that inoculation with *R. intraradices* alleviated the hazardous effects of calcareous soils with high pH on apple rootstock growth [24]. However, how *R. intraradices* affects Cd accumulation and tolerance in *M. hupehensis* Rehd. is completely unknown. In the present study, the effects of AMF on Cd uptake, accumulation, and detoxification in apples were evaluated through a pot experiment in which *M. hupehensis* seedlings were artificially inoculated with *R. intraradices*. Clarifying the physiological and molecular level mechanisms by which *R. intraradices* regulates Cd accumulation, transport, and detoxification in *M. hupehensis* will provide a theoretical basis for reducing the uptake and Cd toxicity in various tree fruit crops.

## 2. Results

### 2.1. Growth Characteristics and Nutrient Element Content

All AMF-inoculated *M. hupehensis* seedlings were successfully colonized with numerous fungal hyphae and arbuscules, with a total colonization rate of 71.3% and 62.0% under Cd-free and Cd stress conditions, respectively (Figure S1a,b). No fungal colonization was detected in the roots of the non-mycorrhizal seedlings (Figure S1a). The colonization rate was significantly decreased in the roots of *M. hupehensis* after Cd exposure (Figure S1b). Cd stress significantly inhibited root growth and activities, whereas AMF inoculation significantly enhanced root activities compared to those of non-mycorrhizal seedlings under Cd treatments (Figure S1c,d). AMF inoculation significantly increased the EE-GRSP and T-GRSP content in soil regardless of Cd treatment (Figure S2).

**Figure 1:**
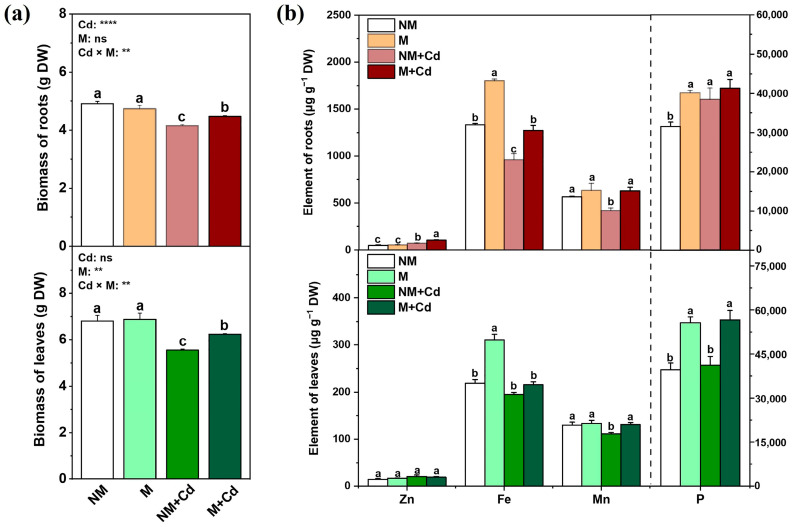
Dry mass (**a**) and nutrient element content (**b**) of roots and leaves of *M. hupehensis* Rehd. without *R. intraradices* inoculation (NM) or with *R. intraradices* inoculation (M) exposed to 0 µM CdCl_2_ or 300 µM CdCl_2_ (+Cd) for 60 d. Data are means ± standard error of the mean (SE; *n* = 3). Different letters on bars indicate significant differences between treatments. *p*-values for ANOVA of CdCl_2_ (Cd), AMF (M), and their interactions are shown. **: *p* ≤ 0.01; ****: *p* ≤ 0.0001; ns: not significant.

In both the presence and absence of AMF inoculation, Cd stress significantly decreased the net photosynthetic rate (Pn), gas conductance (Gs), maximum fluorescence ratio (Fv/Fm), and root and leaf dry mass (DW) compared to those of plants not exposed to Cd (Table 1 and Figure 1a). In response to Cd stress, however, Pn, Gs, Fv/Fm, and the root and leaf DW were significantly increased by 11.9%, 12.8%, 5.1%, 7.7%, and 12.4% in mycorrhizal compared to non-mycorrhizal seedlings (Table 1 and Figure 1a).

Cd stress significantly altered host plant nutrient element uptake, whereas the presence of AMF counteracted these negative effects of Cd exposure (Figure 1b). In the absence of Cd exposure, AMF inoculation significantly increased the root and leaf P and Fe levels relative to those of the uninoculated controls (Figure 1b). Cd exposure significantly increased the root Zn and P but significantly decreased the root Fe and both the root and leaf Mn (Figure 1b). AMF inoculation significantly increased the root Zn, Fe, and Mn and the leaf P and Mn in plants under Cd stress (Figure 1b).

### 2.2. Cd Localization and Accumulation

**Figure 2:**
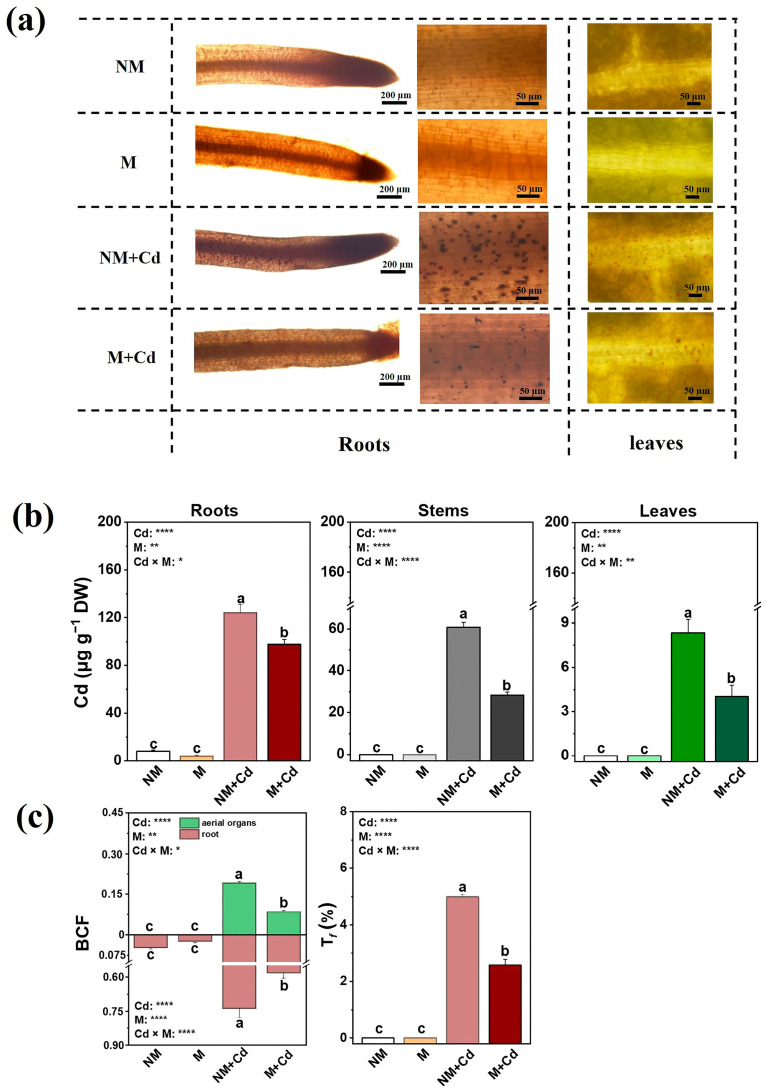
Cd localization (**a**) in the roots and leaves; Cd concentrations (**b**) in the roots, stems, and leaves; and bioconcentration factors (BCFs) of the roots and aerial tissues and translocation factors (T*_f_*) (**c**) for *M. hupehensis* Rehd. without *R. intraradices* inoculation (NM) or with *R. intraradices* inoculation (M) exposed to 0 µM CdCl_2_ or 300 µM CdCl_2_ (+Cd) for 60 d. Data are means ± standard error of the mean (SE; *n* = 3). Different letters on bars indicate significant differences between treatments. *p*-values for ANOVA of CdCl_2_ (Cd), AMF (M), and their interactions are shown. *: *p* ≤ 0.05; **: *p* ≤ 0.01; ****: *p* ≤ 0.0001.

Dithizone histochemical staining was applied to localize Cd in the roots and leaves (Figure 2a). The Cd–dithizone complex was detected in the root cell walls and intercellular spaces of both inoculated and uninoculated *M. hupehensis* exposed to Cd (Figure 2a). Cd was enriched mainly in the leaf veins and several mesophyll cells adjacent to veins (Figure 2a). AMF inoculation significantly reduced Cd deposition in the roots and leaves and Cd accumulation in all tissues compared to the Cd-only treatment (Figure 2b). Thus, the BCFs of root and aerial organs Cd were lower in the mycorrhizal *M. hupehensis* than in the Cd-only treatment (Figure 2c). *T_f_* was 48.2% lower in the AMF-inoculated than in the uninoculated plants exposed to Cd (Figure 2c).

### 2.3. Chemical Forms and Subcellular Distribution of Cd and the Functional Groups of Cell Walls

In the roots of both the inoculated and uninoculated plants, the concentrations and proportions of F_NaCl_ (pectates and protein-integrated Cd) were the highest, followed by F_w_ (water-soluble Cd). F_NaCl_ and F_W_ accounted for 50–64% and 28–36% of the total Cd, respectively (Table 2). Compared with the Cd treatment alone, AMF inoculation decreased the concentrations and proportions of F_E_ (inorganic Cd) and F_w_ but increased the F_NaCl_ proportions in the roots. Cd accumulated mainly in the form of F_HAc_ (undissolved Cd) and F_NaCl_ in the leaves. The concentrations of all Cd forms were significantly lower in the leaves of the AMF-inoculated plants than in those of the non-mycorrhizal controls. However, the proportions of F_NaCl_ and F_HAc_ were significantly higher in the leaves of the inoculated plants exposed to Cd than in those of the uninoculated plants subjected to Cd (Table 2).

Most of the Cd (58–68%) accumulated in the cell walls of the roots and leaves of *M. hupehensis* under Cd stress (Table 3). AMF inoculation lowered the Cd concentration in all subcellular fractions except the root cell walls and vacuoles. In contrast, AMF inoculation significantly increased the proportion of Cd in the root and leaf cell walls but significantly decreased the proportion of Cd in the root plastids and nuclei as well as the root and leaf mitochondria and ribosomes (Table 3).

FTIR analysis was conducted to characterize the functional groups in the root cell walls of *M. hupehensis* subjected to various treatments (Table S1). The A/A_2925_ ratio indicated that the first and second most abundant functional groups in the root cell walls were -OH/-NH and C-O/C-C/C-C-O, respectively, and they were associated with pectin, hemicellulose, and cellulose [25,26,27]. Under Cd stress, the A/A_2925_ values at 3416 cm^−1^, 1078 cm^−1^, and 1024 cm^−1^ in the AMF-inoculated plants were significantly higher than those determined for the uninoculated plants (Table S1).

### 2.4. Phytohormones

**Figure 3:**
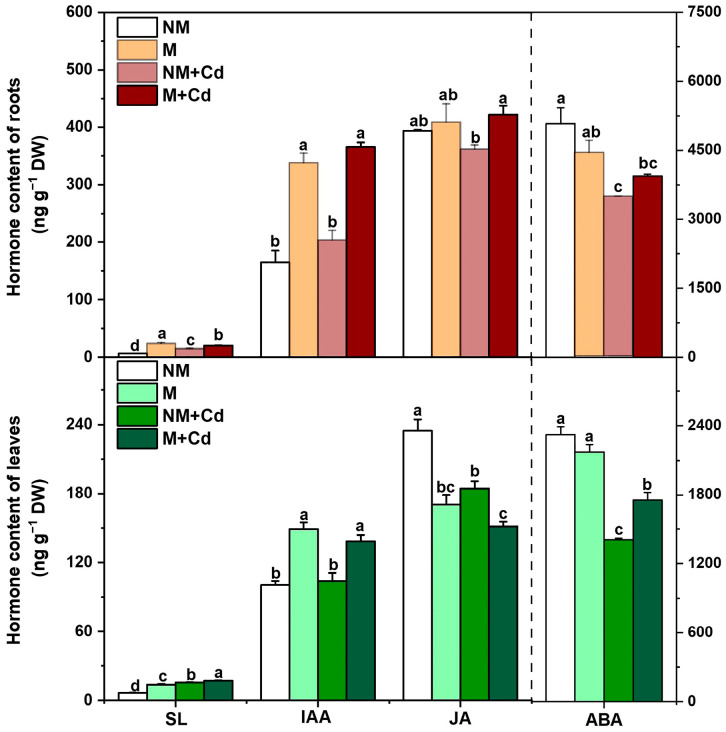
Strigolactone (SL), indoleacetic acid (IAA), jasmonic acid (JA), and abscisic acid (ABA) content in the roots and leaves of *M. hupehensis* Rehd. without *R. intraradices* inoculation (NM) or with *R. intraradices* inoculation (M) exposed to 0 µM CdCl_2_ or 300 µM CdCl_2_ (+Cd) for 60 d. Data are means ± standard error of the mean (SE; *n* = 3). Different letters on bars indicate significant differences between treatments.

Compared with non-mycorrhizal controls, AMF inoculation significantly increased the root strigolactone (SL) and indoleacetic acid (IAA) content but had no significant effect on the root jasmonic acid (JA) content irrespective of Cd treatment (Figure 3). AMF inoculation significantly increased the JA content only in the roots of Cd-exposed plants (Figure 3). While the foregoing trends in SL and IAA content were similar for the leaves, AMF inoculation significantly decreased the leaf JA content regardless of Cd treatment. Under Cd treatment, the abscisic acid (ABA) content was significantly higher in the leaves of the mycorrhizal plants than in those of the non-mycorrhizal plants (Figure 3).

### 2.5. ROS and Antioxidant Systems

**Figure 4:**
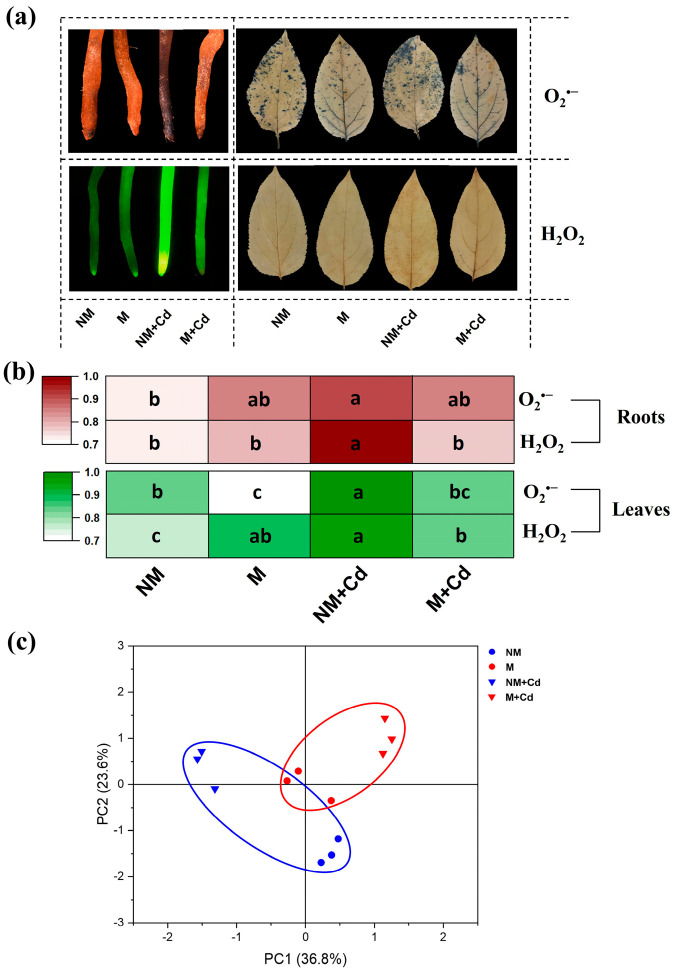
Histochemical detection (**a**) of O_2_^•−^ and H_2_O_2_; O_2_^•−^ and H_2_O_2_ concentrations (**b**); and principal component analysis (PCA) (**c**) of reactive oxygen species (ROS), non-enzymatic antioxidants, and antioxidant enzymes in the roots and leaves of *M. hupehensis* Rehd. without *R. intraradices* inoculation (NM) or with *R. intraradices* inoculation (M) exposed to 0 µM CdCl_2_ or 300 µM CdCl_2_ (+Cd) for 60 d. Data are means ± standard error of the mean (SE; *n* = 3). Values were normalized. Different letters indicate significant differences between treatments.

The root and leaf O_2_^•−^ and H_2_O_2_ concentrations were greater in the Cd-exposed than in the Cd-free non-mycorrhizal plants (Figure 4a,b). AMF inoculation alleviated root and leaf O_2_^•−^ and H_2_O_2_ accumulation in response to Cd exposure (Figure 4a,b).

The root free proline, T-SH, and ascorbate (ASC) levels and the leaf free proline and ASC levels were lower in the non-mycorrhizal *M. hupehensis* subjected to Cd than in those not exposed to Cd (Figure S3). In contrast, Cd exposure increased free proline and soluble phenolics in the roots and all non-enzymatic antioxidants in the leaves of the mycorrhizal plants (Figure S3). Under Cd stress, the concentrations of most non-enzymatic antioxidants except soluble phenolics in the leaves were significantly higher in the roots and leaves of the mycorrhizal plants than in those of the uninoculated plants (Figure S3). The catalase (CAT) and ascorbate peroxidase (APX) activity levels were lower in the roots, while the superoxide dismutase (SOD) activity was higher in the leaves of the non-mycorrhizal plants exposed to Cd than in those of the uninoculated plants not subjected to Cd (Figure S4). However, Cd stress had no significant effects on or increased these antioxidant enzyme activities in the roots and leaves of inoculated plants (Figure S4). The activity levels of most antioxidant enzymes were significantly higher in the mycorrhizal plants than in the non-mycorrhizal plants under Cd stress (Figure S4).

A principal component analysis (PCA) was conducted on the reactive oxygen species (ROS) and nonenzymatic and enzymatic antioxidants to determine the impact of AMF and Cd stress on the redox balance (Figure 4c and Table S2). PC1 separated the effects of Cd, and its contribution was 36.8%. The leaf free proline and ASC and the root CAT substantially contributed to PC1. PC2 separated the effects of AMF, and its contribution was 23.6%. The root O_2_^•−^ and APX and the leaf T-SH and SOD substantially contributed to PC2. The distance between the Cd-free and excess Cd treatments was shorter for the mycorrhizal plants than for the non-mycorrhizal plants. Hence, the redox imbalance resulting from Cd exposure was smaller for the mycorrhizal plants than for the non-mycorrhizal plants.

### 2.6. Transcriptomic Response

**Figure 5:**
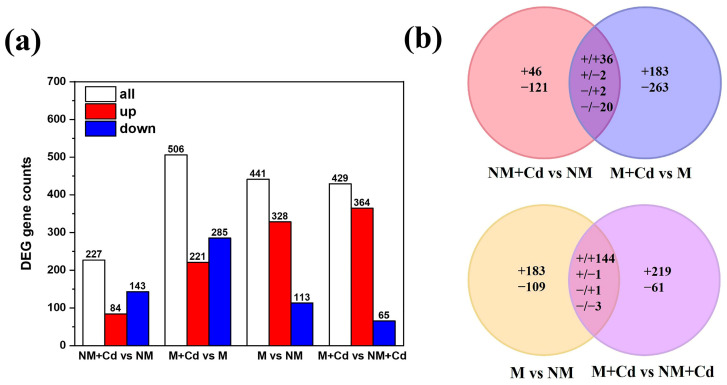
Numbers of upregulated and downregulated differentially expressed genes (DEGs) (**a**) and Venn diagram of DEGs (**b**) in the roots of *M. hupehensis* Rehd. without *R. intraradices* inoculation (NM) or with *R. intraradices* inoculation (M) exposed to 0 µM CdCl_2_ or 300 µM CdCl_2_ (+Cd) for 60 d.

To elucidate the regulatory mechanisms underlying the changes in Cd uptake and accumulation and the physiological responses controlled by AMF, transcriptomes were sequenced in both the non-mycorrhizal and mycorrhizal plants subjected to 0 μM or 300 μM Cd. The sequencing reads are listed in Table S3. A total of 1142 DEGs were screened. Of these, 673 were Cd-responsive and 721 were AMF-responsive. A total of 60 DEGs were common to NM + Cd vs. NM and M + Cd vs. M, while 149 were common to M vs. NM and M + Cd vs. NM + Cd (Figure 5a,b). The mycorrhizal comparison (M + Cd vs. M) showed 122.91% more Cd-responsive DEGs than the non-mycorrhizal comparison (NM + Cd vs. NM). The M + Cd vs. NM + Cd comparison had 12 fewer AMF-responsive DEGs than M vs. NM (Figure 5a). Cd-responsive DEGs showed more downregulation than upregulation regardless of AMF inoculation. In contrast, 546 AMF-responsive DEGs were upregulated, whereas only 173 were downregulated (Figure 5b). To validate the RNA-seq data, we randomly selected 11 genes from the RNA-sequencing readouts for expression verification by RT-qPCR. The gene expression levels largely aligned with the RNA-seq data (Figure S5).

GO and KEGG pathway enrichment analyses were performed to identify the types of DEGs responding to AMF under Cd stress (Figures S6 and S7 and Table S4). The DEGs were assigned to the Biological Process, Molecular Function, and Cellular Component categories for all four treatment comparisons (Figure S6). Strigolactone metabolic process, iron ion binding, and cell wall were the most significantly enriched terms for NM + Cd vs. NM (Figure S6(a1–c1)). Signal transduction, ADP binding, and apoplast were the most significantly enriched terms for M + Cd vs. M (Figure S6(a2–c2)). Signal transduction, cysteine-type peptidase activity, and integral component of plasma membrane were the most significantly enriched terms for M vs. NM (Figure S6(a3–c3)). Lipid catabolic process, cysteine-type peptidase activity, and integral component of plasma membrane were the most significantly enriched terms for M + Cd vs. NM + Cd (Figure S6(a4–c4)). Mycorrhizal plants showed more upregulated DEGs under Biological Process and Molecular Function than non-mycorrhizal plants under Cd stress (Figure S6).

The KEGG pathway enrichment analysis disclosed that Cd stress downregulated relatively more DEGs in the non-mycorrhizal plants than in the mycorrhizal plants (Figure S7(a1,a2)). Nevertheless, AMF inoculation tended to upregulate most DEGs in the roots regardless of Cd treatment (Figure S7(a3,a4)). For the NM + Cd vs. NM group, the downregulated DEGs were enriched mainly in selenocompound metabolism; carotenoid biosynthesis; photosynthesis—antenna proteins; diterpenoid biosynthesis; photosynthesis; and nitrogen metabolism (Figure S7(a1)). For the NM + Cd vs. NM group, the upregulated DEGs were enriched mainly in glycolysis/gluconeogenesis and arginine and tyrosine metabolism (Figure S7(a1)). For the M + Cd vs. M group, the DEGs were enriched mainly in starch and sucrose metabolism; glycolysis/gluconeogenesis; fatty acid degradation; alpha-linolenic acid metabolism tyrosine metabolism; MAPK signaling pathway—plant; and phenylpropanoid biosynthesis (Figure S7(a2)). For the M vs. NM group, the upregulated DEGs were enriched mainly in arginine biosynthesis; alanine, aspartate, and glutamate metabolism; carbon fixation in photosynthetic organisms; tropane, piperidine, and pyridine alkaloid biosynthesis; 2-Oxocarboxylic acid metabolism; phenylalanine metabolism; and isoquinoline alkaloid biosynthesis (Figure S7(a3)). For the M + Cd vs. NM + Cd group, the AMF-responsive upregulated DEGs were enriched in ether lipid metabolism; tyrosine metabolism; phenylalanine, tyrosine, and tryptophan biosynthesis; tropane, piperidine, and pyridine alkaloid biosynthesis; glycerophospholipid metabolism; and arginine biosynthesis and phenylalanine metabolism (Figure S7(a4)). For both M + Cd vs. M and M + Cd vs. NM + Cd, numerous DEGs were enriched in the plant hormone signal transduction pathways (Figure S7).

**Figure 6:**
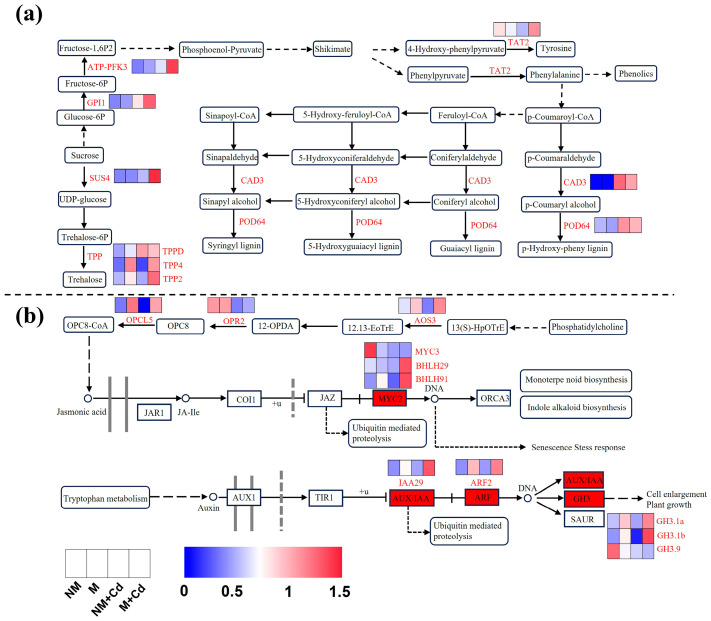
Significantly differentially expressed genes involved in carbohydrate metabolism, amino acid metabolism, and lignin biosynthesis (**a**); and phytohormone signal transduction (**b**) in the roots of *M. hupehensis* Rehd. without *R. intraradices* inoculation (NM) or with *R. intraradices* inoculation (M) exposed to 0 µM CdCl_2_ or 300 µM CdCl_2_ (+Cd) for 60 d. Gradient color barcode represents normalized FPKM values. TPP: trehalose-6-phosphate phosphatase; GPI: glucose-6-phosphate isomerase; SUS: sucrose synthase; ATP-PFK: ATP-dependent 6-phosphofructokinase; TAT: tyrosine aminotransferase; CAD: cinnamyl-alcohol dehydrogenase; POD: peroxidase; AOS: allene oxide synthase; OPR: 12-oxophytodienoate reductase; OPCL: 4-coumarate-CoA ligase-like; MYC: myelocytomatosis; GH: Gretchen Hagen; BHLH: basic helix–loop–helix; IAA: indole-3-acetic acid inducible; ARF: auxin response factor.

In plants, carbohydrate and amino acid biosynthesis and metabolism produce energy and metabolites that alleviate Cd stress [28,29,30]. Here, we identified two pathways related to carbohydrate metabolism, namely starch and sucrose metabolism and glycolysis/gluconeogenesis (Figure 6a). *Trehalose-6-phosphate phosphatase D* (*TPPD*) and *glucose-6-phosphate isomerase 1* (*GPI1*) were upregulated in the Cd treatments (NM + Cd and M + Cd) compared to the Cd-free treatments (NM and M). *Sucrose synthase 4* (*SUS4*), *trehalose-6-phosphate phosphatase 2* (*TPP2*), *TPP4*, and *ATP-dependent 6-phosphofructokinase 3* (*ATP-PFK3*) were significantly upregulated in the mycorrhizal plants compared to the non-mycorrhizal plants under Cd stress (Figure 6a). Cd stress inhibited the expression of *tyrosine aminotransferase2* (*TAT2*), but it was significantly upregulated in mycorrhizal plants compared with those in non-mycorrhizal seedlings under Cd stress (Figure 6a). The lignin biosynthesis-related *cinnamyl-alcohol dehydrogenase 3* (*CAD3*) and *peroxidase 64* (*POD64*) were upregulated in the Cd-treated plants compared to the Cd-free controls regardless of AMF inoculation (Figure 6a).

Phytohormones play critical roles in plant responses to Cd exposure [31]. The JA biosynthesis-related *allene oxide synthase3* (*AOS3*), *12-oxophytodienoate reductase2* (*OPR2*), and *4-coumarate-CoA ligase-like5* (*OPCL5*) were downregulated in the non-mycorrhizal plants exposed to Cd compared with those not subjected to Cd stress (Figure 6b). However, the foregoing genes were significantly upregulated in the AMF-inoculated seedlings under Cd stress (Figure 6b). Cd exposure alone significantly downregulated *MYC3* and *GH3.9,* while AMF inoculation significantly upregulated two *MYC2s* (*BHLH29* and *BHLH91*), one *AUX/IAA* (*IAA29*), one *ARF* (*ARF2*), and two *GH3s* (*GH3.1a* and *GH3.1b*) in the phytohormone signal transduction pathway under Cd stress (Figure 6b).

### 2.7. Transcription of Genes Involved in Cd Uptake, Translocation, and Detoxification

AMF inoculation and Cd treatment affected several DEGs involved in Cd absorption, transport, and tolerance (Figure S5). *ZIP1*, *ZIP5*, and *NRAMP5* regulate root Cd uptake [32,33]. Under Cd stress, *ZIP1.1*, *ZIP1.2*, and *ZIP5* were significantly downregulated, and *NRAMP5* was significantly upregulated in the mycorrhizal seedlings relative to the non-mycorrhizal seedlings (Figure S5). *YSL3* and *HMA2* promote root-to-shoot (acropetal) Cd transport [34,35]. *YSL3.1* and *YSL3.2* were upregulated in the non-mycorrhizal plants exposed to Cd compared to those without Cd stress. Under Cd stress, *YSL3.1* and *YSL3.2* were significantly downregulated, while *HMA2* was significantly upregulated in the mycorrhizal plants compared with the non-mycorrhizal plants (Figure S5). Under Cd stress, MT2 and PDR family gene upregulation improves plant resistance to Cd [36,37]. Under Cd stress, *MT2* was significantly upregulated in the mycorrhizal seedlings compared to the non-mycorrhizal seedlings in the present study. *PDR9* was upregulated in the Cd treatments (NM + Cd and M + Cd) compared to the Cd-free treatments (NM and M). The Cd treatment induced *PDR12* only in the mycorrhizal plants (Figure S5).

## 3. Discussion

### 3.1. Arbuscular Mycorrhizal Fungi Reduce Cd Phytotoxicity and Accumulation in M. hupehensis

The present study demonstrated a high colonization rate for the arbuscular mycorrhizal fungus (AMF) *R. intraradices* in the roots of *M. hupehensis*. Thus, a positive symbiotic relationship (mutualism) existed between *R. intraradices* and *M. hupehensis*. However, cadmium (Cd) exposure significantly reduced AMF colonization in the apple seedlings. This finding was consistent with those of previous studies indicating that high Cd concentrations had negative impacts on AMF spore germination and mycorrhizal colonization [38,39]. GRSP, a special class of glycoprotein, is specifically released by AMF and can chelate heavy metals [40]. Pan et al. [41] found inoculation with *R. aggreratus* decreased Cd bioavailability by increasing the contents of EE-GRSP and T-GRSP, thus reducing Cd uptake in kenaf. Similarly, *R. intraradices* inoculation increased the content of T-GRSP and EE-GRSP, which may be helpful to reduce Cd accumulation in *M. hupehensis*. Cd has no known biological function and is phytotoxic at or above certain threshold concentrations [5]. Here, Cd stress significantly decreased both photosynthetic activity and biomass and upset the nutrient balance in *M. hupehensis*. However, AMF inoculation partially protected *M. hupehensis* against Cd-induced inhibition of tissue biomass, root activity, and photosynthesis, as well as the accumulation of ROS. Hence, AMF inoculation mitigated Cd toxicity in *M. hupehensis*.

Previous study has found that the Cd–dithizone complex was mainly enriched in the surface and the apoplast of root cells [42], which could effectively prevent Cd entering the cell. The similar result was found in both non-mycorrhizal and mycorrhizal *M. hupehensis*. In leaves, Cd was enriched mainly in the leaf veins to decrease direct damage to mesophyll cells. AMF inoculation affects Cd translocation and accumulation in plants. For example, AMF inoculation reduced the Cd concentration in the tissues of *P. yunnanensis* [14], but increased Cd absorption in *Solanum nigrum* [43]. Interspecies differences among plants and fungal inoculants may account for this discrepancy [44]. Here, AMF inoculation significantly reduced Cd–dithizone deposition in the roots and leaves of *M. hupehensis.* This finding was consistent with the observed decreases in the Cd concentrations of the roots and aerial parts, as well as *T_f_*. This mechanism may have enabled the AMF to reduce Cd phytotoxicity and increase Cd tolerance in the host plant [17]. Under Cd stress, the photosynthetic rates were higher, the tissue biomass was greater, and the ROS levels were lower in the inoculated than in the uninoculated *M. hupehensis*. To date, however, little has been reported about the physiological and molecular mechanisms by which AMF regulate Cd accumulation and phytotoxicity in *Malus* spp.

### 3.2. AMF Reduce Cd Mobility in M. hupehensis

The phytotoxicity of Cd varies with its chemical form. As inorganic Cd (F_E_) and water-soluble Cd (F_W_) are highly mobile in plants, they are more phytotoxic than the relatively less mobile pectate- and protein-chelated Cd (F_NaCl_) and oxalate Cd (F_HCl_) [45,46]. The present study showed that AMF inoculation decreased the proportions of F_E_ and F_W_ and increased those of F_NaCl_ and F_HCl_ in *M. hupehensis* roots and leaves. Zhang et al. [17] reported that AMF improved Cd tolerance in *Zea mays* by increasing its inactive Cd content. The mobility and toxicity of Cd also depend on its subcellular compartmentalization [47]. Here, Cd was most highly concentrated in the cell walls of *M. hupehensis* roots and leaves. The functional groups in the cell wall serve as ligands that trap and immobilize Cd ions via adsorption, ion exchange, and precipitation. Thus, the cell wall is a first-pass defense against Cd phytotoxicity [48,49,50]. AMF inoculation induced a distinct subcellular Cd distribution. The present study increased the Cd proportion in the cell walls, thereby decreasing the Cd concentrations in root and leaf cell organelles. Wang et al. [51] reported similar discoveries for *Medicago sativa* L. inoculated with *Glomus intraradices*.

The AMF-inoculated plants showed clear shifts in functional groups in the cell wall, especially at 3416 cm^−1^, 1078 cm^−1^, and 1024 cm^−1^. Wu et al. [52] found that the higher abundance of –OH at 3416 cm^−1^ in the cell walls of *Brassica napus* root contributed to the cell wall binding of Cd. In the present study, AMF inoculation induced supplementary –OH group production in the root cell walls of *M. hupehensis,* thereby increasing its Cd tolerance. The bands near 1078 cm^−1^ and 1024 cm^−1^ were attributed to the C–O/C–C/C-C-O stretching vibrations of cellulose and hemicellulose [26,53]. Gao et al. [18] stated that AMF inoculation enhanced C–O/C–C abundance in the root cell walls of rice under Cd stress. This mechanism increased cell wall resistance to Cd phytotoxicity. Under Cd stress, AMF inoculation enhanced Cd immobilization by increasing the abundance of functional groups in the cell walls.

### 3.3. AMF Facilitate the Physiological Mitigation of Cd Phytotoxicity in M. hupehensis

AMF inoculation increases plant Cd tolerance through several physiological mechanisms. Improving mineral absorption by AMF inoculation is one of the most frequently reported mechanisms to help plants overcome HM toxicity [54]. Here, AMF inoculation significantly increased the Zn, Fe, and Mn content in the roots and the P and Mn content in the leaves of *M. hupehensis*. It also increased the Zn, Fe, and Mn content in *Medicago sativa* [8] and the P and Fe content in kenaf (*Hibiscus cannabinus*) [41] under Cd stress. AMF-mediated increases in nutrient elements such as Zn and Fe compete against the uptake of non-essential, toxic Cd [55]. Thus, plant-mycorrhizal symbiosis confers protection against abiotic stressors such as heavy metal-contaminated soils through the AMF-mediated maintenance of ion homeostasis [14].

Endogenous hormones play a key role in plant development, but they are also closely related to the abiotic stress tolerance of plants [56]. AMF increase the production of phytohormones such as SL, IAA, JA, and ABA, which confer resistance to abiotic stress including drought, salt, and low temperature in host plants by coordinating different signal transduction pathways [57,58,59,60]. SL induces spore germination and promotes hyphal growth in AMF [61]. Application of the strigolactone GR24 improved Cd tolerance in *Hordeum vulgare* L. by regulating its Cd uptake and antioxidant metabolism [62]. The relatively high SL content in mycorrhizal *M. hupehensis* may reduce Cd phytotoxicity by regulating Cd accumulation and scavenging Cd-induced ROS. AMF increase the biomass of *M. hupehensis* under Cd stress, possibly by increasing both the root and the leaf IAA content which is consistent with *Medicago sativa* [8]. The JA level of the host plant may affect the AMF colonization rate and arbuscule formation [57]. Tomato plants defective in JA synthesis showed lower AMF root colonization rates than their wild-type counterparts [63]. In the present study, the increase in JA content in the mycorrhizal *M. hupehensis* under Cd stress may have strengthened the mutualism between AMF and the host plants. Applying appropriate concentrations of JA can inhibit the expression of Cd absorption and transport genes [31], increase Cd compartmentalization in cell walls, promote antioxidant enzyme biosynthesis, and alleviate Cd-mediated growth inhibition [47] in plants. Applying appropriate concentrations of ABA limited Cd uptake in *P. euphratica* [64] and *Vigna radiata* [65] and increased the resistance of plants to Cd by enhancing their antioxidant capacity [66]. AMF increase the ABA levels in host plants under abiotic stress [67]. Relatively high root and leaf ABA levels were associated with higher plant Cd tolerance in mycorrhizal *M. hupehensis* under Cd stress.

AMF inoculation also alleviated Cd phytotoxicity in alfalfa plants by increasing their GSH, free proline, and ASC levels [8]. It also enhanced the antioxidant enzyme activity in *Lonicera japonica* [68] and *P. yunnanensis* [14]. Here, we found that AMF inoculation increased free proline, soluble phenolics, T-SH, and ASC and induced SOD, CAT, and APX in *M. hupehensis*, thereby augmenting its ability to scavenge Cd-induced ROS and attenuate Cd phytotoxicity.

### 3.4. Molecular Mechanism by Which AMF Reduce Cd Phytotoxicity in M. hupehensis

Transcriptomic analysis revealed that AMF increased Cd resistance in *M. hupehensis* by altering the expression levels of Cd tolerance-related genes. This discovery aligned with the findings reported for poplar by Liu et al. [14]. Here, most of the DEGs corresponding to Cd and AMF were enriched in the carbohydrate metabolism, amino acid biosynthesis and metabolism, and phytohormone biosynthesis and signal transduction pathways. Trehalose is a non-reducing sugar that protects certain cellular structures against oxidative stress [69]. AMF inoculation upregulated the trehalose biosynthesis-related genes *SUS* and *TPP* in response to Cd stress. Thus, it promoted trehalose biosynthesis under Cd stress. Zhou et al. [28] found that upregulation of the glycolysis/gluconeogenesis metabolism pathway genes produced the substances and energy required to reduce Cd-induced phytotoxicity in *Brassica parachinensis* under Cd stress. Here, Cd exposure further upregulated *GPI1* and *ATP-PFK3*, which are involved in the glycolysis/gluconeogenesis pathway. Therefore, AMF may alleviate Cd stress by enhancing carbohydrate metabolism in *M. hupehensis*.

The KEGG enrichment analysis indicated that several DEGs in the amino acid biosynthesis and metabolism pathways were significantly upregulated in the mycorrhizal plants compared to the non-mycorrhizal plants under Cd stress. Free amino acids have negatively charged hydroxyl and carboxyl groups that chelate metal ions, enhance antioxidant capacity, and reduce Cd phytotoxicity in plants [70]. For example, certain Compositae [71], *Arabidopsis thaliana*, and tomato [72] mitigate Cd-induced oxidative damage by increasing their tyrosine (Tyr) and/or tryptophan (Trp) content. Abiotic stress activates the phenylalanine (Phe) metabolic pathway, which in turn initiates the biosynthesis of phenolic compounds with antioxidant capacity that enable plants to scavenge ROS [73]. The arginine (Arg) and proline metabolic pathways generate osmoprotectants that resist the osmotic imbalances caused by Cd stress. This mechanism improved Cd tolerance in *Sedum plumbizincicola* [74]. Similarly, AMF reduced Cd phytotoxicity in *M. hupehensis* by regulating free amino acid biosynthesis and metabolism. Lignin, a major plant cell wall component, inhibits the migration of heavy metal cations and their entry into the cytoplasm [75]. Here, the Cd treatment significantly upregulated lignin biosynthesis-related genes in both the inoculated and the uninoculated *M. hupehensis*, thereby augmenting the ability of the plants to contend with Cd stress.

The present study also disclosed that several genes related to the JA biosynthesis and the JA and IAA signal transduction pathways were significantly upregulated in the mycorrhizal seedlings under Cd stress. Therefore, JA also enables the plant to cope with Cd stress. Gu et al. [76] reported similar observations for maize. In the present study, AMF may have increased Cd tolerance in the apple seedlings by upregulating the genes in the glycerophospholipid metabolism and peroxisome pathways. Certain glycerophosphate metabolites help to stabilize cell membranes against destructive stressors [77]. Peroxisomes are involved in photorespiration, phytohormone biosynthesis, and ROS metabolism and rapidly scavenge ROS [78].

AMF decrease root uptake and acropetal translocation in *M. hupehensis*, possibly by regulating the transcription of genes controlling Cd absorption and transport. Certain *ZIP*, *YSL*, and *MT* genes regulate Cd uptake, transport, and detoxification [5]. AMF inoculation also affects plant Cd uptake by regulating the expression of ZIP and NRAMP family genes [19,79]. For example, *F. mosseae* can promote Cd uptake in *Phragmites australis* by upregulating the expression of *ZIP* and *NRAMP* genes [19]. On the contrary, *G. versiforme* inoculation led to a significant downregulation of *NRAMP5* in upland rice roots, thus reducing Cd absorption and accumulation [79]. Here, AMF inoculation lowered the relative *ZIP1.1*, *ZIP1.2*, and *ZIP5* mRNA levels and, by extension, Cd accumulation in *M. hupehensis* roots. The YSL gene family mediates the root-to-shoot transport of metal cation–nicotinamide chelates [80]. Enhanced root-to-shoot Cd transport was observed in *Arabidopsis thaliana* overexpressing *YSL3* from wheat [35]. The present study showed that AMF inoculation downregulated root *YSL3* in *M. hupehensis* under Cd stress. This observation was consistent with the fact that mycorrhizal *M. hupehensis* had lower aerial organ Cd translocation and accumulation rates than uninoculated plants. Metallothioneins (MTs) are low-molecular-weight proteins that chelate heavy metal cations and lower their phytotoxicity [81]. Zhu et al. [79] demonstrated that AMF may reduce Cd phytotoxicity in rice by upregulating its *MTs*. Here, *MT2* was upregulated in the mycorrhizal *M. hupehensis* under Cd stress. Hence, this gene may be essential to Cd detoxification in woody fruit tree species.

## 4. Materials and Methods

### 4.1. Plant Material, Treatment, and Sample Collection

The experiments were conducted in a greenhouse at Shenyang Agricultural University, Shenyang, China (41°49′ N, 123°341′ E). *Malus hupehensis* seeds were surface-disinfected with 5% (*v*/*v*) H_2_O_2_ for 10 min and rinsed with sterile distilled water. The seeds were then stratified at 0–4 °C for 40 d, germinated, and sown in nursery plates containing autoclaved substrate. The plants were then grown in a greenhouse under natural light and temperature conditions (28 °C day/18 °C night and 50–60% relative humidity) for 30 d. As shown in Figure S8, seedlings ~8 cm in height were transferred to 16 cm × 16 cm plastic pots, each containing 1 kg of an autoclaved 1:1 *v*/*v* soil–sand mixture, and randomly divided into two groups. The soil characteristics were as follows: pH 6.01, 9.62 g kg^−1^ organic matter, 22.12 mg kg^−1^ available nitrogen, 34.85 mg kg^−1^ available phosphorus, and 51.63 mg kg^−1^ available potassium. The first group included seedlings inoculated with 10 g *R. intraradices* (each gram of inoculum contained approximately 60 spores), while the second included plants treated with inoculum inactivated by autoclaving at 121 °C for 2 h. The AMF was purchased from the Beijing Academy of Agriculture and Forestry, Beijing, China. After 30 d, 50 mL of distilled water or 300 μM CdCl_2_ was irrigated to the plants of each group in every day. The concentration of Cd treatment used in the present study was selected on the basis of a previous study [82]. Hence, there were four treatments in total: seedlings uninoculated (NM) or inoculated with the AMF (M) grown on Cd-free substrate or 300 μM Cd (+Cd) stress conditions. One seedling was cultured per pot. Each treatment had three replicates with six plants each. Plants were harvested after Cd treatment for 60 d.

Mycorrhizal colonization rates were determined according to the method of Huang et al. [83]. Root samples were randomly selected from four treatments, cut into 1 cm sections, soaked in 10% (*w*/*v*) KOH in a 90 °C water bath for 30 min, soaked in 10% (*v*/*v*) H_2_O_2_ at room temperature for 10 min, soaked in 2% (*v*/*v*) HCl for 30 min, stained with 0.05% (*w*/*v*) trypan blue at 90 °C for 20 min, and decolorized several times with 1:1:1 (*v*/*v*/*v*) lactic acid/glycerol/water. One hundred stained subsamples were randomly selected and examined under a microscope to calculate the mycorrhizal colonization rate according to the method of Wu and Xia [84]. The contents of easily extractable glomalin-related soil protein (EE-GRSP) and total glomalin-related soil protein (T-GRSP) were extracted and determined according to the method of Zhang et al. [85].

### 4.2. Plant Physiological Parameter Measurements

Photosynthetic parameters including the net photosynthetic rate (Pn), transpiration rate (Tr), and stomatal conductance (Gs) were determined using a CIRAS-2 photosynthesis system (PP Systems, Amesbury, MA, USA) according to our previous approach: Zhou et al. [7]. After 30 min of dark adaptation, chlorophyll fluorescence values (Fv/Fm—maximum efficiency of PSII) were measured by a portable fluorometer (Pocket PEA, Hansatech, UK) [86].

The distribution and accumulation of O_2_^•−^ and H_2_O_2_ in the fine roots and leaves were detected based on the histochemical method [87]. The concentrations of O_2_^•−^ and H_2_O_2_ in the roots and leaves were determined with a spectrophotometer as described by He et al. [88].

The concentrations of free proline and soluble phenolics were determined spectrophotometrically using the approach described by He et al. [88]. The total thiols (T-SH) and ascorbate (ASC) levels were measured quantitatively according to the method described previously [89]. The soluble proteins were extracted from roots and leaves and used for the measurement of enzyme activities, as suggested previously [90]. The enzyme activities of superoxide dismutase (SOD) and ascorbate peroxidase (APX) were determined spectrophotometrically following the method of Chen et al. [91], and that of catalase (CAT) following Ma and Cheng [92]. 

### 4.3. Determination of Cd Localization and Concentration and Nutrient Elements

Cd localization in the roots and leaves of plants was investigated using the histochemical staining method suggested by He et al. [87]. In brief, root and leaf tissues were rinsed in deionized H_2_O and exposed to a staining solution (15 mg diphenylthiocarbazone dissolved in 30 mL acetone, 10 mL H_2_O, and 50 μL glacial acetic acid) for 30 min. After a brief rinse in deionized H_2_O, the well-stained samples with Cd–dithizone precipitates were photographed under a light microscope (Eclipse E200; Nikon, Tokyo, Japan) using a DS-Fi1 CCD camera (Nikon).

Root, stem, and leaf fine powder samples (0.3 g) were weighed and digested with a mixture of HNO_3_ and HClO_4_ (7:1, *v*:*v*) at 170 °C as suggested by Ma et al. [89]. The concentrations of Cd, Zn, Fe, and Mn were determined by a flame atomic absorbance spectrometry (Hitachi 180-80, Hitachi Ltd., Tokyo, Japan). Plant P concentrations were determined using the molybdate-blue colorimetric method after digestion [93]. The translocation factor (*T_f_*) and bioconcentration factor (BCF) was calculated according to He et al. [87].

### 4.4. Subcellular Distribution and Chemical Forms of Cd

Subcellular distribution of Cd in the root and leaf tissues was determined after separating subcellular fractions by the sucrose-gradient centrifugation technique following a previous method [94]. The chemical forms of Cd in the roots and leaves were determined by flame atomic absorbance spectrometry (Hitachi 180-80, Hitachi Ltd., Tokyo, Japan) according to our previous approach [7].

### 4.5. Analysis of Characteristic Functional Groups of Root Cell Walls

CWs of M. hupehensis roots were separated and purified as suggested by Chen et al. [95]. The functional group in the root cell wall was characterized by Fourier-transform infrared spectroscopy (FTIR) spectroscopy (VERTEX 70; Bruker Corp., Billerica, MA, USA) as previously described [18]. In total, 2 mg of lyophilized root cell wall was mixed with 200 mg potassium bromide (KBr) and pressed into thin slices. The functional groups in cell walls were identified by an FTIR spectroscope within the 400–4000 cm^−1^ scanning range and at a 4 cm^−1^ resolution.

### 4.6. Determination of Phytohormone

The extraction and purification of endogenous strigolactone (SL), indole acetic acid (IAA), jasmonic acid (JA), and abscisic acid (ABA) levels were performed according to the method described previously [96]. Then, according to the manufacturer’s instructions, the endogenous hormones were analyzed by an ELISA kit (Jiangsu Meimian Industrial Co., Ltd., Jiangsu, China).

### 4.7. Transcriptome Sequencing and q-PCR Validation of Genes

RNA extraction, library preparation, and sequencing were conducted according to the procedures of Huang et al. [97]. Plant total RNA was extracted from 12 root samples with the RNAprep Pure Plant Kit (Tiangen, Beijing, China). Then, libraries were constructed using total RNA with excellent quality and integrity. The libraries were sequenced on an Illumina NovaSeq 6000 Platform (Illumina, San Diego, CA, USA). After removing the raw data containing adapter and poly-N and those with low quality, the clean reads were then mapped to the GDDH13v1.1 reference genome (https://www.rosaceae.org/species/malus/malus_x_domestica/genome_GDDH13_v1.1 (accessed on 10 June 2024)) using Hisat2 Tools (version 2.2.1) (https://github.com/DaehwanKimLab/hisat2 (accessed on 10 June 2024)). The gene expression levels in fragments per kilobase of transcript per million fragments mapped (FPKM) were estimated. Differential gene expression analyses of both treatment groups were performed using DESeq2 (version 1.30.1) (https://bioconductor.org/packages/release/bioc/html/DESeq2.html (accessed on 14 June 2024)). Genes with an adjusted fold change (FC) ≥ 2 and a false discovery rate (FDR) < 0.05 were designated as differentially expressed. Gene Ontology (GO) and Kyoto Encyclopedia of Genes and Genomes (KEGG) enrichment analyses of the DEGs were performed using the clusterProfiler package (version 4.4.4) [98,99]. The raw RNA-seq dataset was submitted to the Sequencing Read Archive (SRA; National Center for Biotechnology Information (NCBI, Bethesda, MD, USA, https://www.ncbi.nlm.nih.gov/, accessed on 6 November 2024)) under accession No. PRJNA1184943 (https://www.ncbi.nlm.nih.gov/sra/PRJNA1184943).

For q-PCR validation, total RNA extraction, RNA reverse transcription, and quantitative PCR were performed according to a previously described method [100]. The gene-specific primers are listed in Table S5, and *β*-*actin* was the reference gene. Relative gene expression levels were calculated using the 2^−ΔΔCt^ method [101]. Expression levels were set to unity for each gene in the roots of *M. hupehensis* treated with 0 μM Cd and without AMF inoculation (NM). The corresponding transcript FCs were calculated for the other two treatments.

### 4.8. Statistical Analysis

All data were first tested for normality using Statgraphics (version 18) (https://www.statgraphics.com/ (accessed on 10 June 2024)). Two-way analyses of variance (ANOVAs) were applied using the 300 μM Cd (Cd) and AMF (M) treatments as the main factors, and all parameters were tested for statistically significant change. All experimental results were presented as means ± standard error of the mean (SE). All *p*-values obtained from the multiple comparisons were corrected by the Tukey–HSD method to reduce the chance of type I errors. Differences between means were considered statistically significant at *p* < 0.05.

## 5. Conclusions

**Figure 7:**
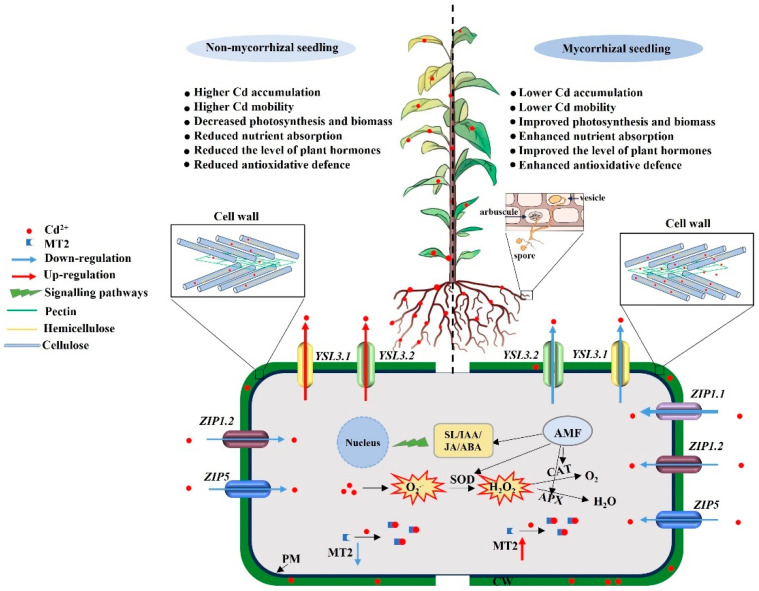
Schematic model of AMF-enhanced Cd tolerance in *M. hupehensis* Rehd. exposed to 0 µM CdCl_2_ or 300 µM CdCl_2_ (+Cd) for 60 d. Cd^2+^ uptake, accumulation and detoxification in non-mycorrhizal seedling (left) and mycorrhizal seedling (right). Mycorrhizal seedlings exhibited lower root Cd accumulation and root-to-shoot translocation capacity but higher antioxidant defense activity, resulting in lower growth reduction than non-mycorrhizal seedlings. ZIP1.1, ZIP1.2, and ZIP5: zinc/iron-regulated transporter-related proteins 1.1, 1.2, and 5; YSL3.1 and YSL3.2: yellow stripe-like transporter 3.1 and 3.2; MT2: metallothionein 2.

As summarized in Figure 7, Cd was absorbed and accumulated in various tissues of *Malus hupehensis*, inhibited photosynthesis, reduced biomass, impaired nutrient element homeostasis, and triggered ROS production. Arbuscular mycorrhizal fungus (AMF; *R. intraradices*) inoculation enhanced nutrient element uptake and reduced Cd accumulation and acropetal translocation in *M. hupehensis*. AMF inoculation promoted Cd binding to cell walls, reduced organellar Cd content, and lowered Cd mobility. It also mitigated Cd-induced oxidative stress injury by increasing the content of non-enzymatic antioxidants such as free proline, soluble phenolics, T-SH, and ASC and the activity of antioxidant enzymes including SOD, CAT, and APX. Transcriptomics analysis revealed that under Cd stress, AMF inoculation upregulated DEGs in the carbohydrate metabolism, amino acid biosynthesis and metabolism, and phytohormone biosynthesis and signal transduction pathways, thereby mitigating damage to *M. hupehensis* caused by Cd. AMF inoculation also decreased and increased Cd uptake and tolerance, respectively, in *M. hupehensis* under Cd stress by modulating the expression levels of the DEGs associated with Cd uptake, transport, and tolerance. The results obtained from this study will provide a theoretical basis for reducing heavy metal absorption and increasing heavy metal tolerance in fruit trees grown in heavy metal contaminated soil.

## Figures and Tables

**Table 1:** Net photosynthetic rate (Pn; µmol CO_2_ m^−2^ s^−1^), stomatal conductance (Gs; mmol H_2_O m^−2^ s^−1^), transpiration rate (Tr; mmol H_2_O m^−2^ s^−1^), and maximum quantum efficiency of leaves of *M. hupehensis* Rehd. without *R. intraradices* inoculation (NM) or with *R. intraradices* inoculation (M) exposed to 0 µM CdCl_2_ or 300 µM CdCl_2_ (+Cd) for 60 d. Data are means ± standard error of the mean (SE; *n* = 3). Different letters after values indicate significant differences between treatments. *p*-values for ANOVA of CdCl_2_ (Cd), AMF (M), and their interactions are shown. **: *p* ≤ 0.01; ****: *p* ≤ 0.0001; ns: not significant.

Treatments	Pn	Gs	Tr	Fv/Fm
NM	14.40 ± 0.64 a	225.50 ± 2.60 b	3.70 ± 0.06 b	0.83 ± 0.01 a
M	14.20 ± 0.12 a	253.00 ± 5.77 a	4.40 ± 0.06 a	0.83 ± 0.01 a
NM + Cd	10.55 ± 0.09 c	153.00 ± 1.15 d	3.90 ± 0.01 b	0.78 ± 0.01 c
M + Cd	11.80 ± 0.12 b	172.50 ± 8.37 c	3.90 ± 0.12 b	0.82 ± 0.01 b
*P*_(Cd)_	****	****	ns	****
*P*_(M)_	ns	**	**	****
*P*_(Cd×M)_	ns	ns	**	****

**Table 2:** Chemical forms of Cd and their relative proportions in the roots and leaves of *M. hupehensis* Rehd. without *R. intraradices* inoculation (NM) or with *R. intraradices* inoculation (M) exposed to 300 µM CdCl_2_ (+Cd) for 60 d. Data are means ± standard error of the mean (SE; *n* = 3). Different letters after values indicate significant differences between treatments. F_E_: inorganic Cd (extracted by 80% ethanol); F_w_: water-soluble Cd (extracted by deionized water); F_NaCl_: pectates and protein-integrated Cd (extracted by 1 M NaCl); F_HAc_: undissolved Cd (extracted by 2% HAC); F_HCl_: oxalate Cd (extracted by 0.6 M HCl).

Tissue	Treatment	Cd Concentration (μg g^−1^ DW)					Percentage of Cd in Different Chemical Forms (%)				
		F_E_	F_w_	F_NaCl_	F_HAc_	F_HCl_	F_E_	F_w_	F_NaCl_	F_HAc_	F_HCl_
Root	NM + Cd	7.23 ± 0.15 a	39.91 ± 1.41 a	57.20 ± 5.23 a	5.03 ±0.75 a	4.09 ± 0.49 a	6.38 ± 0.16 a	35.29 ± 1.91 a	50.24 ± 3.03 b	4.44 ± 0.63 a	3.65 ± 0.58 a
	M + Cd	1.90 ± 0.23 b	26.62 ± 2.15 b	58.80 ± 4.77 a	3.07 ± 0.29 a	2.32 ± 0.04 b	2.06 ± 0.24 b	28.69 ± 0.29 b	63.35 ± 0.54 a	3.36 ± 0.44 a	2.54 ± 0.21 a
Leaf	NM + Cd	0.80 ± 0.00 a	1.25 ± 0.03 a	2.10 ± 0.14 a	2.89 ± 0.12 a	0.37 ± 0.04 a	10.83 ± 0.38 a	16.89 ± 0.80 a	28.33 ± 1.12 b	39.01 ± 1.02 b	4.95 ± 0.31 a
	M + Cd	0.26 ± 0.01 b	0.41 ± 0.01 b	1.14 ± 0.05 b	1.49 ± 0.03 b	0.16 ± 0.02 b	7.58 ± 0.52 b	11.90 ± 0.15 b	32.87 ± 0.94 a	43.02 ± 0.12 a	4.64 ± 0.65 a

**Table 3:** Subcellular distribution of Cd and its relative proportions in the roots and leaves of *M. hupehensis* Rehd. without *R. intraradices* inoculation (NM) or with *R. intraradices* inoculation (M) exposed to 300 µM CdCl_2_ (+Cd) for 60 d. Data are means ± standard error of the mean (SE; *n* = 3). Different letters after values indicate significant differences between treatments.

Tissue	Treatment	Cd Concentration (μg g^−1^ DW)						Percentage of Cd in Different Chemical Forms (%)					
		Cell Wall	Plastid	Nucleus	Mitochondrion	Ribosome	Vacuole	Cell Wall	Plastid	Nucleus	Mitochondrion	Ribosome	Vacuole
Root	NM + Cd	71.91 ± 3.61 a	2.96 ± 0.18 a	3.96 ± 0.10 a	3.84 ± 0.38 a	3.22 ± 0.13 a	30.21 ± 2.01 a	62.09 ± 0.69 b	2.55 ± 0.06 a	3.21 ± 0.19 a	3.32 ± 0.29 a	2.80 ± 0.21 a	26.04 ± 0.53 a
	M + Cd	64.08 ± 2.81 a	1.30 ± 0.22 b	1.47 ± 0.21 b	1.20 ± 0.08 b	1.37 ± 0.23 b	25.64 ± 1.69 a	67.45 ± 0.52 a	1.37 ± 0.21 b	1.54 ± 0.20 b	1.28 ± 0.14 b	1.43 ± 0.18 b	26.93 ± 0.48 a
Leaf	NM + Cd	4.02 ± 0.21 a	0.31 ± 0.02 a	0.36 ± 0.04 a	0.34 ± 0.02 a	0.34 ± 0.01 a	1.52 ± 0.06 a	58.27 ± 1.08 b	4.54 ± 0.22 a	5.13 ± 0.36 a	4.87 ± 0.17 a	5.02 ± 0.26 a	22.17 ± 1.47 a
	M + Cd	2.35 ± 0.05 b	0.16 ± 0.01 b	0.16 ± 0.01 b	0.12 ± 0.02 b	0.09 ± 0.01 b	0.08 ± 0.07 b	63.96 ± 1.45 a	4.28 ± 0.21 a	4.26 ± 0.29 a	3.20 ± 0.53 b	2.53 ± 0.13 b	21.76 ± 1.11 a

## Data Availability

The data used to support the findings of this study are available from the author upon request.

## Supplementary Materials

The following supporting information can be downloaded at https://www.mdpi.com/article/10.3390/ijms26041418/s1.
