# Supplementary material for: Arbuscular Mycorrhizal Fungi Alleviate Cadmium Phytotoxicity by Regulating Cadmium Mobility, Physiological Responses, and Gene Expression Patterns in Malus hupehensis Rehd
Source: Int J Mol Sci. 2025 Feb 7;26(4):1418. doi: 10.3390/ijms26041418 (PMC11855468; doi:10.3390/ijms26041418)
Supplement: Supplementary file 1 [file ijms-26-01418-s001.zip › Supplementary Material-Figures.pdf]

Arbuscular Mycorrhizal Fungi Alleviate Cadmium Phytotoxicity by Regulating Cadmium Mobility, Physiological Responses, and Gene Expression Patterns in *Malus hupehensis* Rehd

Xiaolei Zhuang [1,2], Siyu Liu [1,2], Shengzhe Xu [1], Sijun Qin [1,2], Deguo Lyu [1,2], Jiali He [1,2,*] and Jiangtao Zhou [3,*]

[1] College of Horticulture, Shenyang Agricultural University, Shenyang 110866, China; zhuangxiaolei0701@163.com (X.Z.); siyuliu1119@163.com (S.L.); xushengzhe0234@163.com (S.X.); qsj1975@syau.edu.cn (S.Q.); lvdeguo@163.com (D.L.)

[2] Key Lab of Fruit Quality Development and Regulation of Liaoning Province, Shenyang 110866, China

[3] Research Institute of Pomology, Chinese Academy of Agricultural Sciences, Xingcheng 125100, China

* Correspondence: hejiali1017@syau.edu.cn (J.H.); zhoujiangtao@caas.cn (J.Z.); Tel.: +86-24-8848-7143 (J.H.)

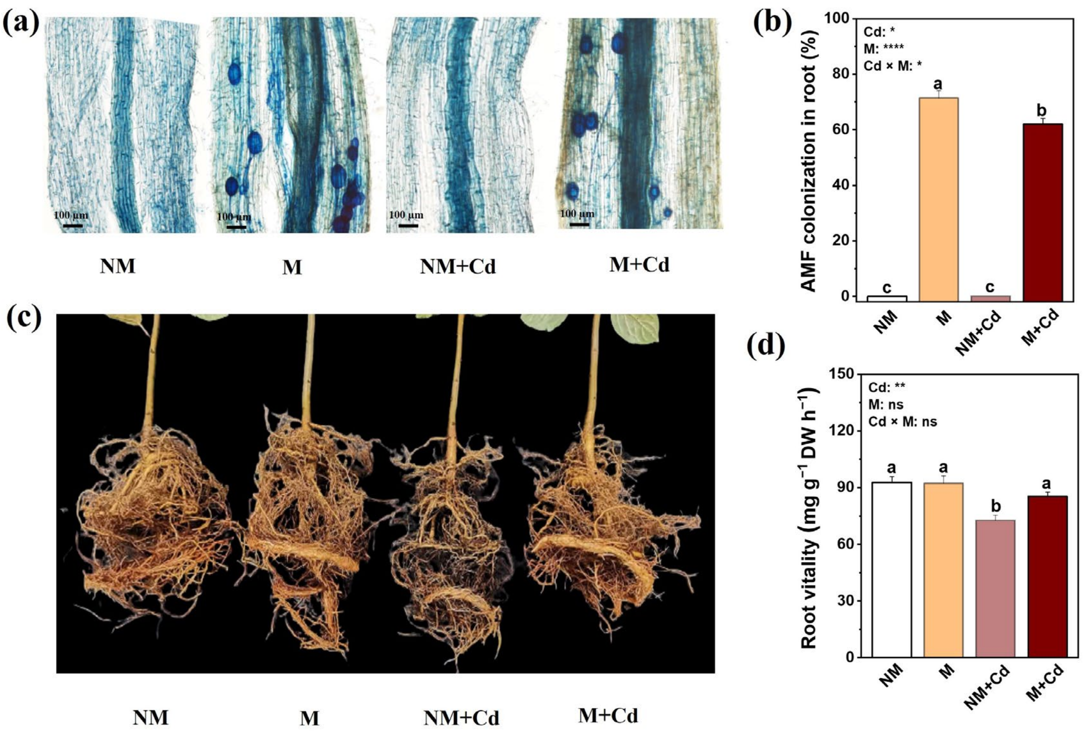

Figure S1. Roots of uninoculated and inoculated *M. hupehensis* Rehd. plants (a). AMF colonization rates (b), root growth status (c), and root vitality (d) of *M. hupehensis* Rehd. without *R. intraradices* inoculation (NM) or with *R. intraradices* inoculation (M) exposed to 0 μM $CdCl_2$ or 300 μM $CdCl_2$ (+Cd) for 60 d. Data are means ± standard error of the mean (SE; n = 3). Different letters on bars indicate significant differences between treatments. P-values for ANOVA of $CdCl_2$ (Cd), AMF (M), and their interactions are shown. *: $P \leq 0.05$; **: $P \leq 0.01$; ****: $P \leq 0.0001$; ns: not significant.

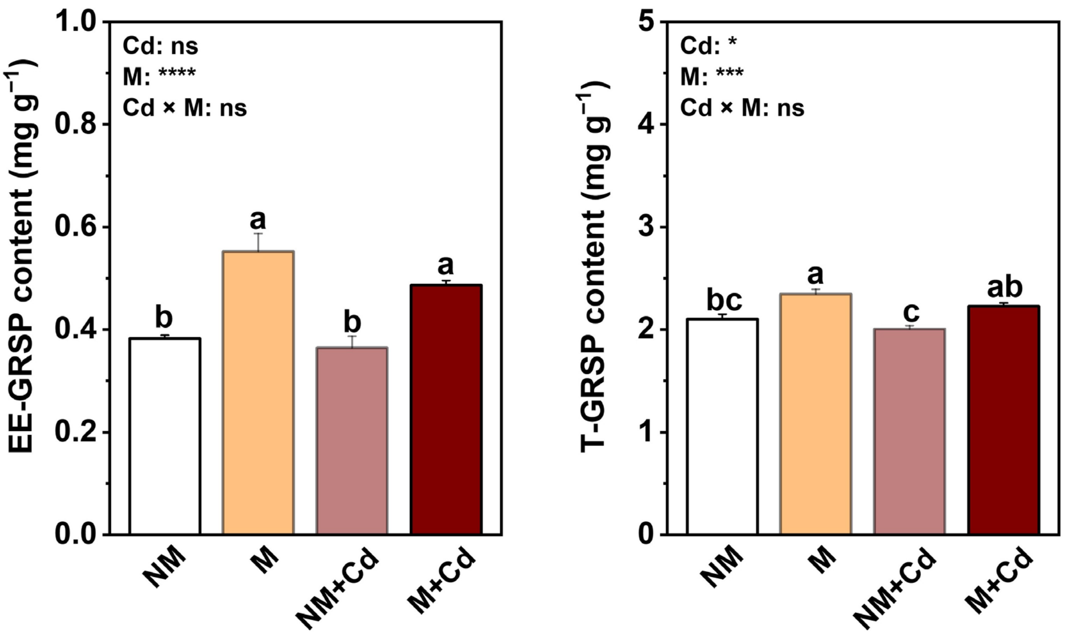

Figure S2. The contents of EE-GRSP and T-GRSP in soil under different treatments.

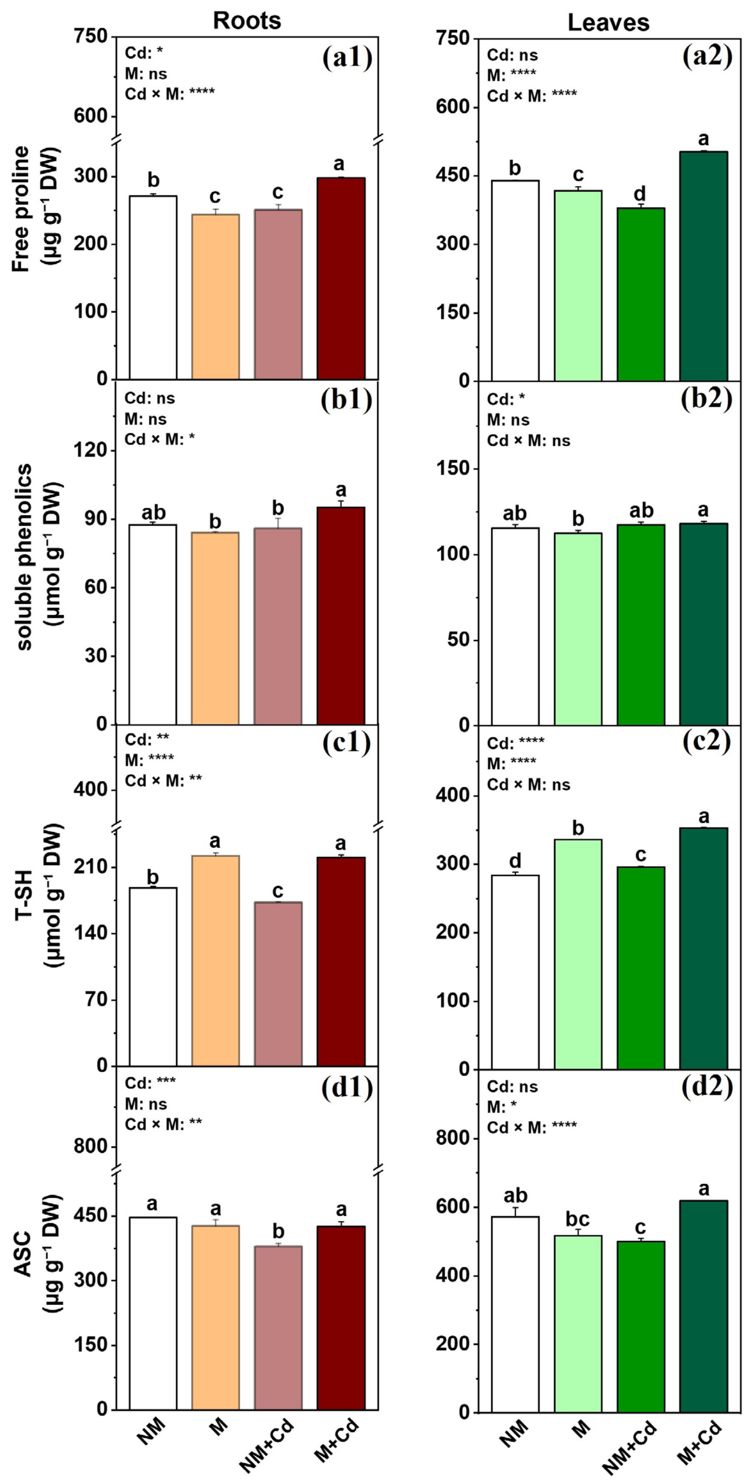

Figure S3. Free proline (a1-a2), soluble phenolics (b1-b2), T-SH (c1-c2), and ASC (d1-d2) in the root (a1-d1) and leaf (a2-d2) of *M. hupehensis* Rehd. without *R. intraradices* inoculation (NM) or with *R. intraradices* inoculation (M) exposed to 0 μM $CdCl_2$ or 300 μM $CdCl_2$ (+Cd) for 60 d. Data are means ± standard error of the mean (SE; n = 3). Different letters on bars indicate significant differences between treatments. P-values for ANOVA of $CdCl_2$ (Cd), AMF (M), and their interactions are shown. *: $P \leq 0.05$; **: $P \leq 0.01$; ***: $P \leq 0.001$; ****: $P \leq 0.0001$; ns: not significant.

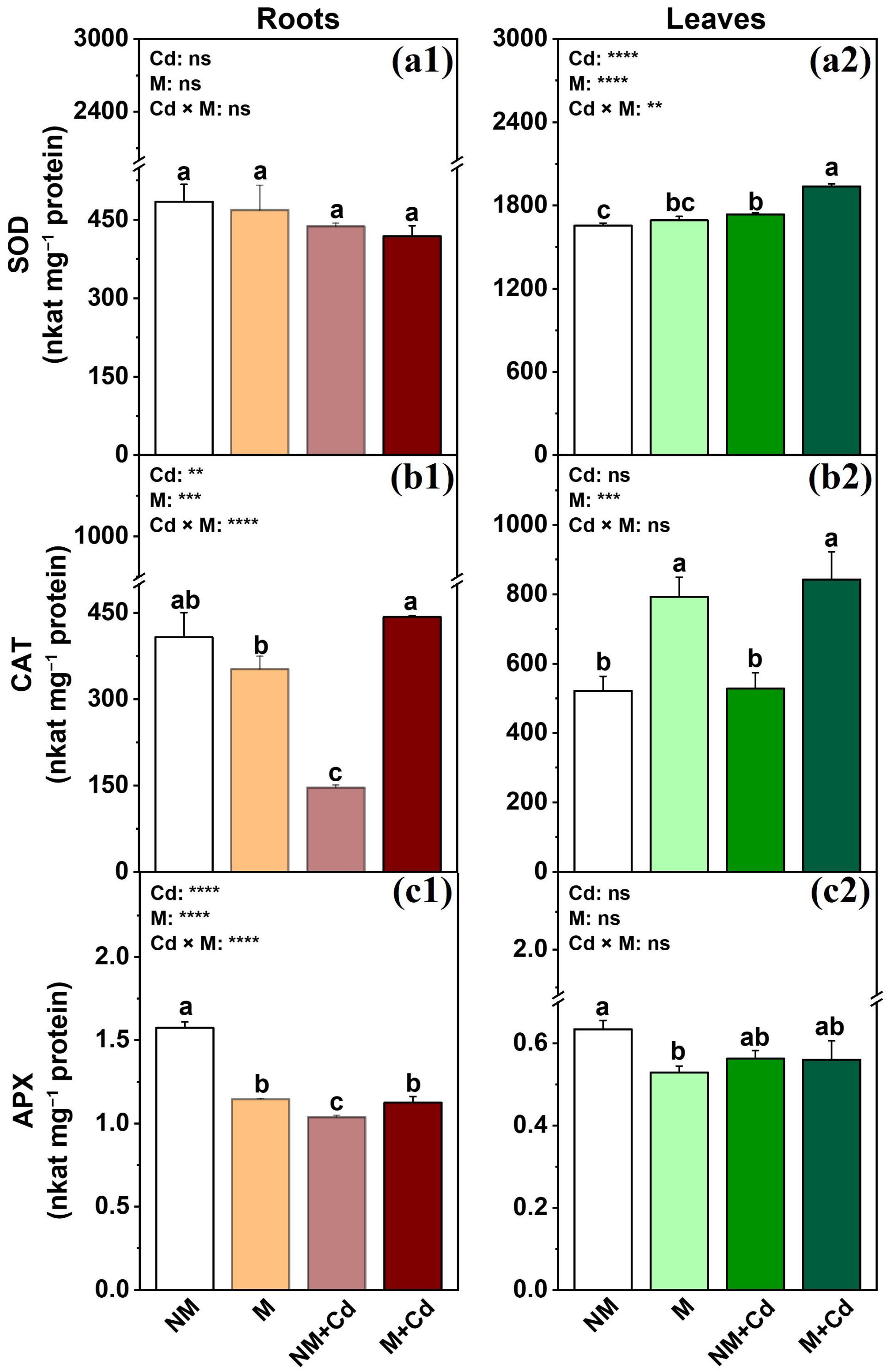

Figure S4. SOD (a1-a2), CAT (b1-b2), and APX (c1-c2) in the root (a1-c1) and leaf (a2-c2) of *M. hupehensis* Rehd. without *R. intraradices* inoculation (NM) or with *R. intraradices* inoculation (M) exposed to 0 μM $CdCl_2$ or 300 μM $CdCl_2$ (+Cd) for 60 d. Data are means ± standard error of the mean (SE; n = 3). Different letters on bars indicate significant differences between treatments. *P*-values for ANOVA of $CdCl_2$ (Cd), AMF (M), and their interactions are shown. **: $P \leq 0.01$; ***: $P \leq 0.001$; ****: $P \leq 0.0001$; ns: not significant.

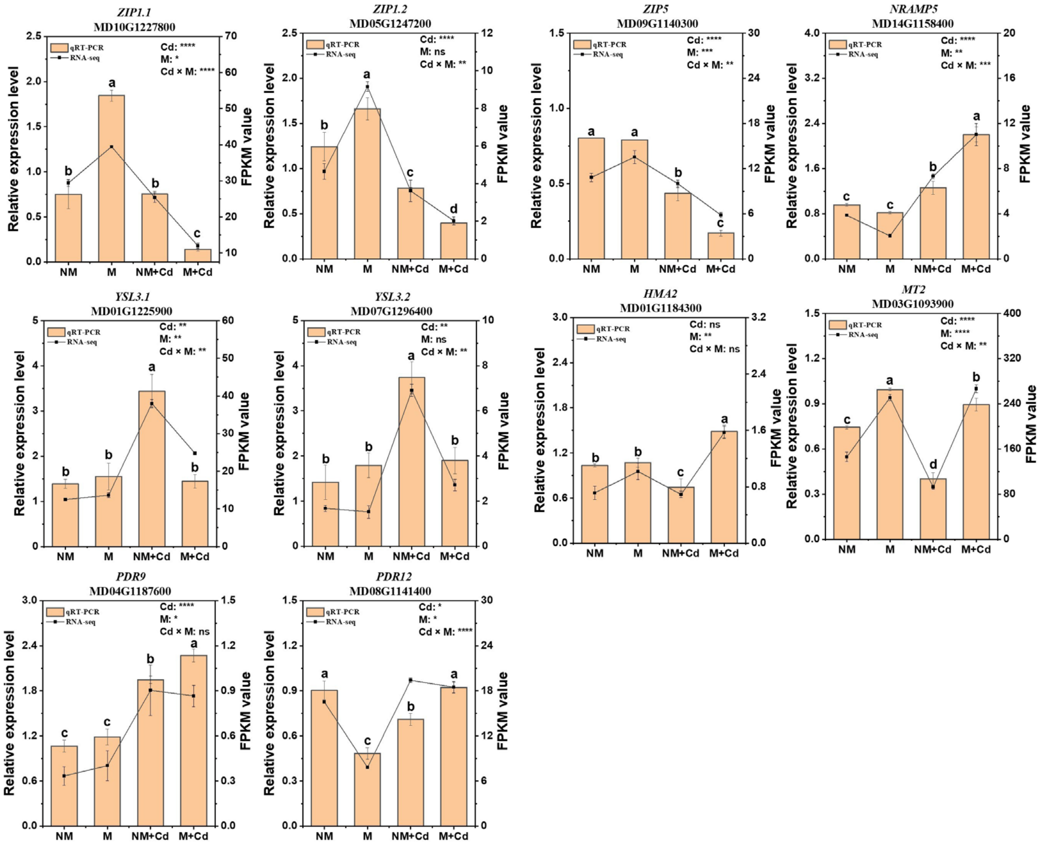

Figure S5. Relative gene expression of eleven DEGs in the roots by qRT-PCR and RNA sequencing.

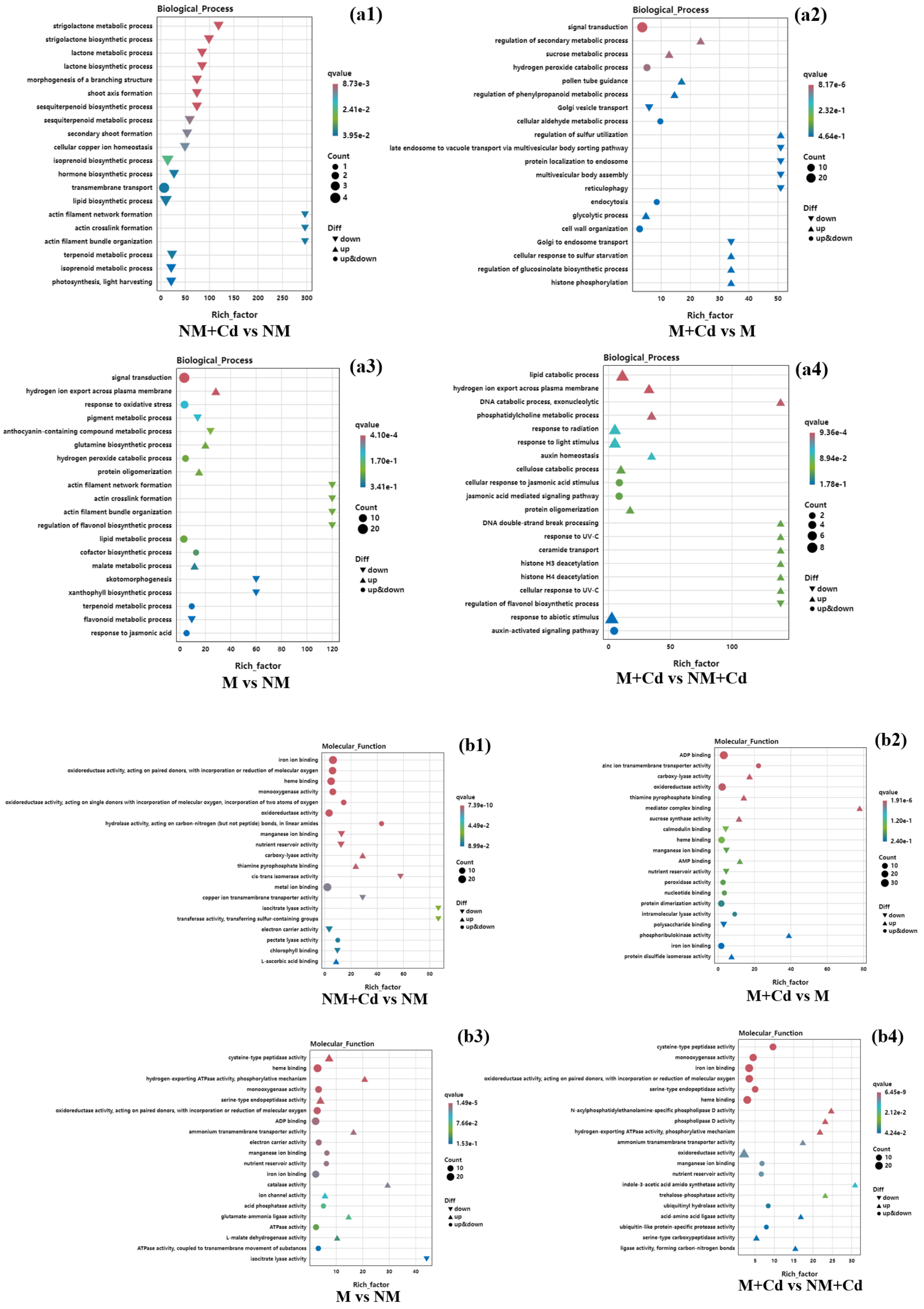
(a1)
Biological_Process
strigolactone metabolic process
strigolactone biosynthetic process
lactone metabolic process
lactone biosynthetic process
morphogenesis of a branching structure
shoot axis formation
sesquiterpenoid biosynthetic process
sesquiterpenoid metabolic process
secondary shoot formation
cellular copper ion homeostasis
isoprenoid biosynthetic process
hormone biosynthetic process
transmembrane transport
lipid biosynthetic process
actin filament network formation
actin crosslink formation
actin filament bundle organization
terpenoid metabolic process
isoprenoid metabolic process
photosynthesis, light harvesting
qvalue
8.73e-3
2.41e-2
3.95e-2
Count
1
2
3
4
Diff
▼ down
▲ up
● up&down
Rich_factor
NM+Cd vs NM
(a2)
Biological_Process
signal transduction
regulation of secondary metabolic process
sucrose metabolic process
hydrogen peroxide catabolic process
pollen tube guidance
regulation of phenylpropanoid metabolic process
Golgi vesicle transport
cellular aldehyde metabolic process
regulation of sulfur utilization
late endosome to vacuole transport via multivesicular body sorting pathway
protein localization to endosome
multivesicular body assembly
reticulophagy
endocytosis
glycolytic process
cell wall organization
Golgi to endosome transport
cellular response to sulfur starvation
regulation of glucosinolate biosynthetic process
histone phosphorylation
qvalue
8.17e-6
2.32e-1
4.64e-1
Count
10
20
Diff
▼ down
▲ up
● up&down
Rich_factor
M+Cd vs M
(a3)
Biological_Process
signal transduction
hydrogen ion export across plasma membrane
response to oxidative stress
pigment metabolic process
anthocyanin-containing compound metabolic process
glutamine biosynthetic process
hydrogen peroxide catabolic process
protein oligomerization
actin filament network formation
actin crosslink formation
actin filament bundle organization
regulation of flavonol biosynthetic process
lipid metabolic process
cofactor biosynthetic process
malate metabolic process
skotomorphogenesis
xanthophyll biosynthetic process
terpenoid metabolic process
flavonoid metabolic process
response to jasmonic acid
qvalue
4.10e-4
1.70e-1
3.41e-1
Count
10
20
Diff
▼ down
▲ up
● up&down
Rich_factor
M vs NM
(a4)
Biological_Process
lipid catabolic process
hydrogen ion export across plasma membrane
DNA catabolic process, exonucleolytic
phosphatidylcholine metabolic process
response to radiation
response to light stimulus
auxin homeostasis
cellulose catabolic process
cellular response to jasmonic acid stimulus
jasmonic acid mediated signaling pathway
protein oligomerization
DNA double-strand break processing
response to UV-C
ceramide transport
histone H3 deacetylation
histone H4 deacetylation
cellular response to UV-C
regulation of flavonol biosynthetic process
response to abiotic stimulus
auxin-activated signaling pathway
qvalue
9.36e-4
8.94e-2
1.78e-1
Count
2
4
6
8
Diff
▼ down
▲ up
● up&down
Rich_factor
M+Cd vs NM+Cd
(b1)
Molecular_Function
iron ion binding
oxidoreductase activity, acting on paired donors, with incorporation or reduction of molecular oxygen
heme binding
monooxygenase activity
oxidoreductase activity, acting on single donors with incorporation of molecular oxygen, incorporation of two atoms of oxygen
oxidoreductase activity
hydrolase activity, acting on carbon-nitrogen (but not peptide) bonds, in linear amides
manganese ion binding
nutrient reservoir activity
carboxy-lyase activity
thiamine pyrophosphate binding
cis-trans isomerase activity
metal ion binding
copper ion transmembrane transporter activity
isocitrate lyase activity
transferase activity, transferring sulfur-containing groups
electron carrier activity
pectate lyase activity
chlorophyll binding
L-ascorbic acid binding
qvalue
7.39e-10
4.49e-2
8.99e-2
Count
10
20
Diff
▼ down
▲ up
● up&down
Rich_factor
NM+Cd vs NM
(b2)
Molecular_Function
ADP binding
zinc ion transmembrane transporter activity
carboxy-lyase activity
oxidoreductase activity
thiamine pyrophosphate binding
mediator complex binding
sucrose synthase activity
calmodulin binding
heme binding
manganese ion binding
AMP binding
nutrient reservoir activity
peroxidase activity
nucleotide binding
protein dimerization activity
intramolecular lyase activity
polysaccharide binding
phosphoribulokinase activity
iron ion binding
protein disulfide isomerase activity
qvalue
1.91e-6
1.20e-1
2.40e-1
Count
10
20
30
Diff
▼ down
▲ up
● up&down
Rich_factor
M+Cd vs M
(b3)
Molecular_Function
cysteine-type peptidase activity
heme binding
hydrogen-exporting ATPase activity, phosphorylative mechanism
monooxygenase activity
serine-type endopeptidase activity
oxidoreductase activity, acting on paired donors, with incorporation or reduction of molecular oxygen
ADP binding
ammonium transmembrane transporter activity
electron carrier activity
manganese ion binding
nutrient reservoir activity
iron ion binding
catalase activity
ion channel activity
acid phosphatase activity
glutamate-ammonia ligase activity
ATPase activity
L-malate dehydrogenase activity
ATPase activity, coupled to transmembrane movement of substances
isocitrate lyase activity
qvalue
1.49e-5
7.66e-2
1.53e-1
Count
10
20
Diff
▼ down
▲ up
● up&down
Rich_factor
M vs NM
(b4)
Molecular_Function
cysteine-type peptidase activity
monooxygenase activity
iron ion binding
oxidoreductase activity, acting on paired donors, with incorporation or reduction of molecular oxygen
serine-type endopeptidase activity
heme binding
N-acylphosphatidylethanolamine-specific phospholipase D activity
phospholipase D activity
hydrogen-exporting ATPase activity, phosphorylative mechanism
ammonium transmembrane transporter activity
oxidoreductase activity
manganese ion binding
nutrient reservoir activity
indole-3-acetic acid amido synthetase activity
trehalose-phosphatase activity
ubiquitinyl hydrolase activity
acid-amino acid ligase activity
ubiquitin-like protein-specific protease activity
serine-type carboxypeptidase activity
ligase activity, forming carbon-nitrogen bonds
qvalue
6.45e-9
2.12e-2
4.24e-2
Count
10
20
Diff
▼ down
▲ up
● up&down
Rich_factor
M+Cd vs NM+Cd

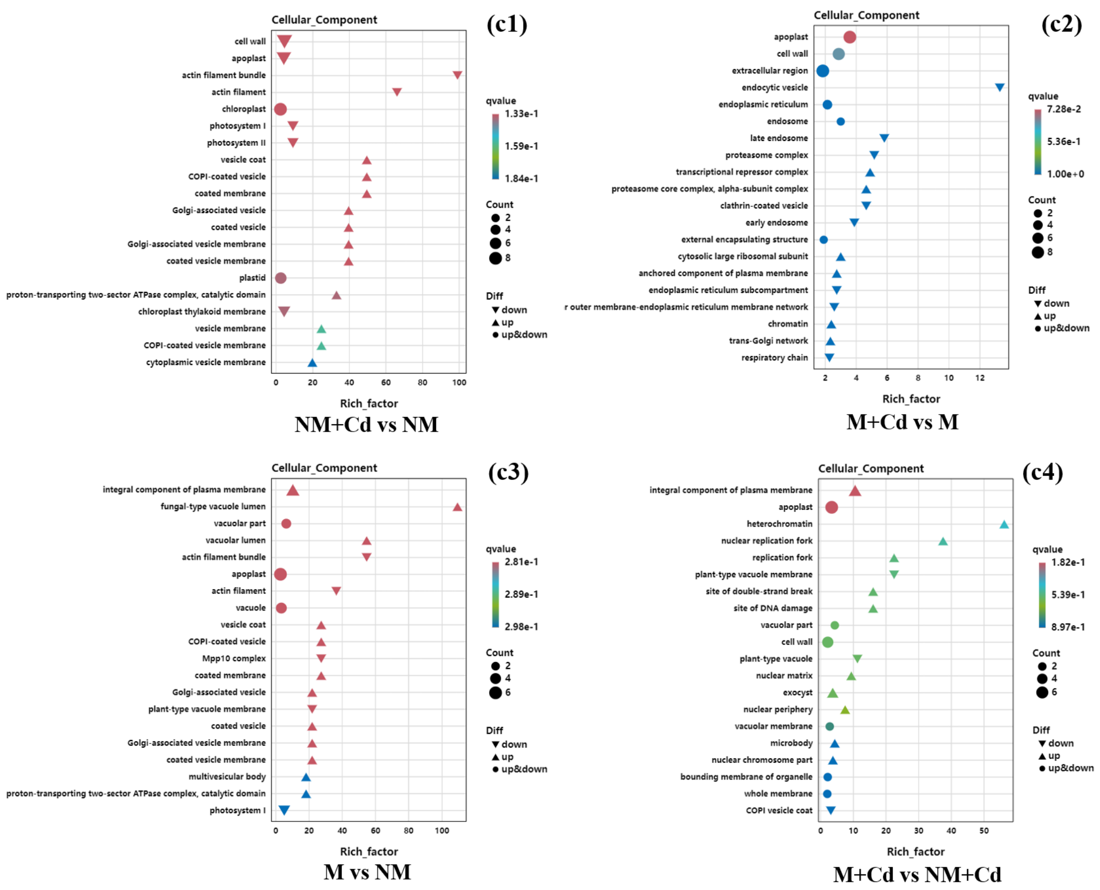

Figure S6. The top 20 Gene Ontology (GO) term (a1-a4, Biological Process; b1-b4, Molecular Function; c1-c4, Cellular Component) enrichment analysis of all annotated differential expression genes (DEGs) in roots of *M. hupehensis* Rehd. without *R. intraradices* inoculation (NM) or with *R. intraradices* inoculation (M) exposed to 0 μM $CdCl_2$ or 300 μM $CdCl_2$ (+Cd) for 60 d.

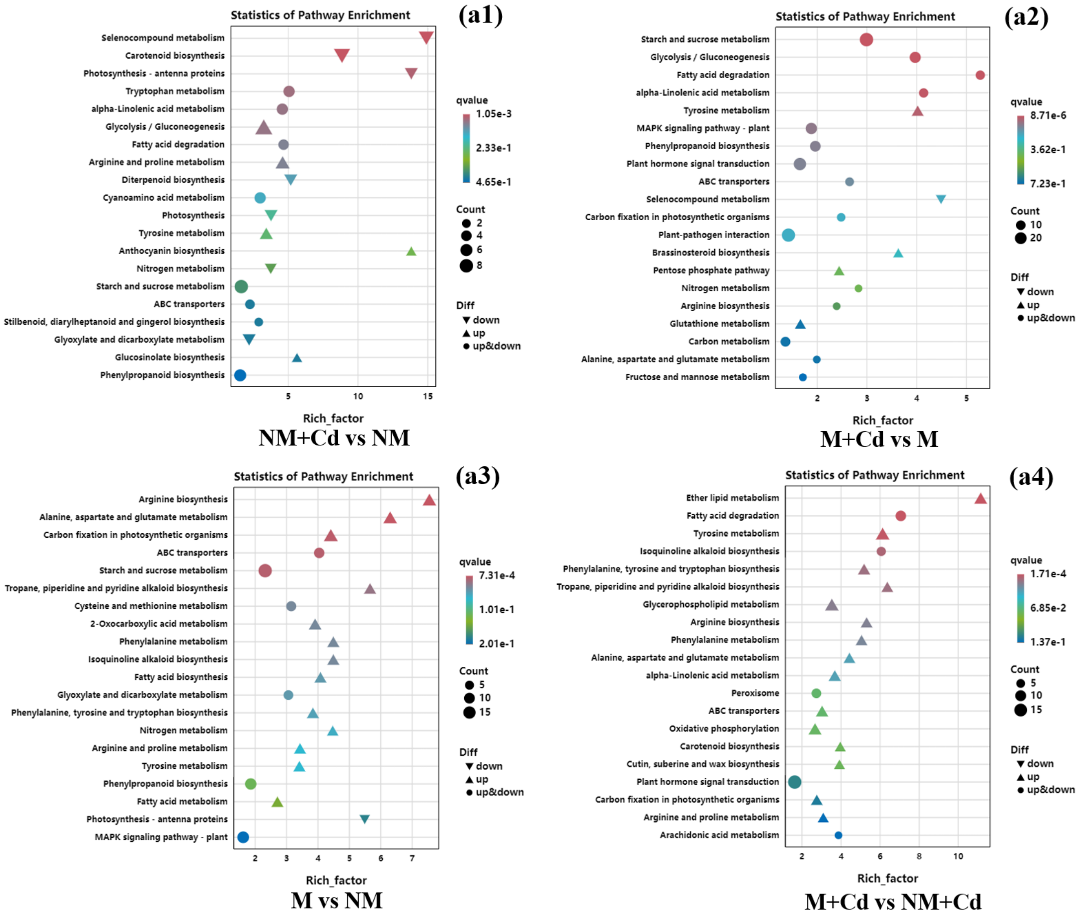

Figure S7. The top 20 gene Kyoto Encyclopedia of Genes and Genomes (KEGG) pathway enrichment analysis of all annotated differential expression genes (DEGs) in roots of *M. hupehensis* Rehd. without *R. intraradices* inoculation (NM) or with *R. intraradices* inoculation (M) exposed to 0 μM $CdCl_2$ or 300 μM $CdCl_2$ (+Cd) for 60 d.

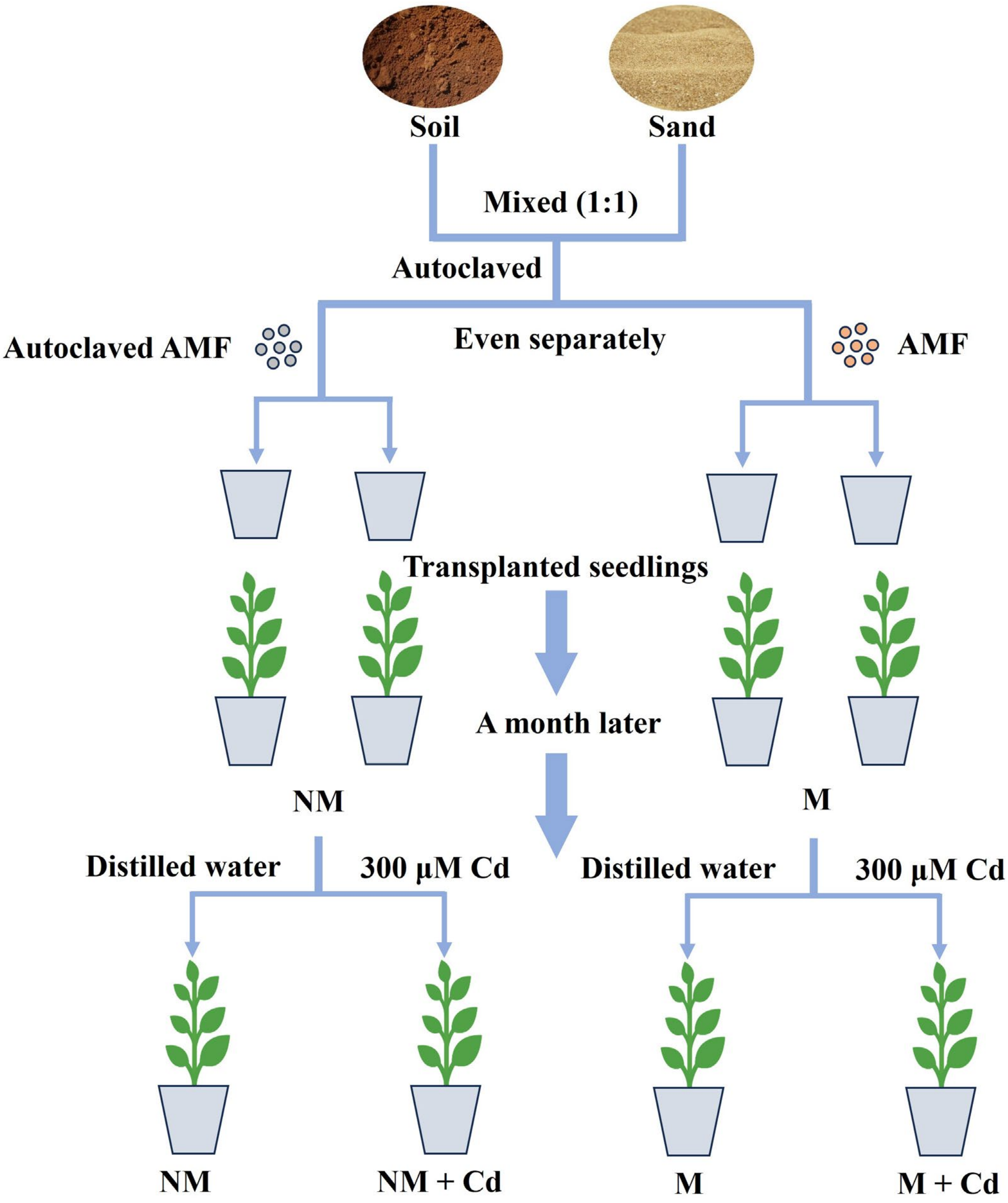

Figure S8. The schematic of *M. hupehensis* Rehd. without *R. intraradices* inoculation (NM) or with *R. intraradices* inoculation (M) exposed to 0 µM $CdCl_2$ or 300 µM $CdCl_2$ (+Cd) for 60 d.
